# A numerical approach for preventing the dispersion of infectious disease in a meeting room

**DOI:** 10.1038/s41598-022-21161-z

**Published:** 2022-10-10

**Authors:** Mahdi Ahmadzadeh, Mehrzad Shams

**Affiliations:** Faculty of Mechanical Engineering, K. N. Toosi University of Technology, Pardis St., Vanak Sq., Tehran, Iran

**Keywords:** Infectious diseases, Sustainability, Civil engineering, Mechanical engineering

## Abstract

Airborne transmission of respiratory aerosols carrying infectious viruses has generated many concerns about cross-contamination risks, particularly in indoor environments. ANSYS Fluent software has been used to investigate the dispersion of the viral particles generated during a coughing event and their transport dynamics inside a safe social-distance meeting room. Computational fluid dynamics based on coupled Eulerian–Lagrangian techniques are used to explore the characteristics of the airflow field in the domain. The main objective of this study is to investigate the effects of the window opening frequency, exhaust layouts, and the location of the air conditioner systems on the dispersion of the particles. The results show that reducing the output capacity by raising the concentration of suspended particles and increasing their traveled distance caused a growth in the individuals' exposure to contaminants. Moreover, decreasing the distance between the ventilation systems installed location and the ceiling can drop the fraction of the suspended particles by over 35%, and the number of individuals who are subjected to becoming infected by viral particles drops from 6 to 2. As well, the results demonstrated when the direction of input airflow and generated particles were the same, the fraction of suspended particles of 4.125%, whereas if the inputs were shifted to the opposite direction of particle injection, the fraction of particles in fluid increased by 5.000%.

## Introduction

Since late 2019 and to this day, the world community has faced an invisible enemy, which has considerably affected the various aspects of sustainable development of societies and raised concerns regarding the influence of indoor environment flow dynamics on human health. Social distancing, sanitization, wearing masks, avoidance of the recirculation of air, and the use of air purifier systems to clean the indoor air have been identified and recommended as efficient prevention strategies^1,2^. Nevertheless, some concerns and challenges still exist in ensuring healthy indoor environment conditions due to many uncertainties regarding the COVID-19 transmission routes. One of the important questions that have emerged during this pandemic is how the heating, ventilation, and air conditioning (HVAC) systems affect the distribution of the virus in indoor environments. To achieve this importance, simulating the virus-laden droplet distribution and spreading generated by the respiratory events of an infected person in indoor environments is essential. Because the experimental study is challenging and costly, computational fluid dynamic (CFD) provides a valuable and reliable tool for evaluating this issue^3,4^.

Several studies are available in the literature regarding the flow-dynamic behavior of COVID-19 in different environments. The main mechanisms for the spread of pathogenic agents are respiratory activities in infected persons, such as speaking, coughing, and sneezing, which can be transmitted via aerosols, droplets, fomites, and feces, affecting the health of people^5–8^. In a numerical study, Liu et al.^9^ investigated the airborne transmission route through coughing and sneezing under dry and humid conditions for saliva droplets. Their results showed that under dry ambient conditions, the number of airborne potentially virus-laden nuclei increased more than four times compared to wet ambient conditions. Specification of the initial velocity of respiratory expelled particles poses a colorful role in numerical modeling. The experimental measurement results have cleared that the initial velocity range of coughed droplets is different between 6 and 22 m/s, and most of the numerical studies have introduced and used 11.2 m/s as an average velocity for coughing^10,11^.

Another important factor that affects the transport chain and dispersion of contaminated particles released during indoor respiratory events is their size^12,13^. These droplets and aerosols are distributed in a wide size range. Experimental results showed that depending on the type of event and the characteristics of the emission source, the droplet size can vary from 0.1 to 1000 μm^10,14,15^. The difference in particle size can cause differences concerning their trajectories in the air. This diversity of behavior is due to the different balances between the forces acting on the particles in air transport. Gravity and aerodynamic forces are the main forces that are considered to act on such particles^14^. The relationship between these forces strongly depends on the particle sizes. For equivalent particle diameters smaller than 10 µm, the aerodynamic forces are more important than the force of gravity, and thus, the particles are floating and follow the airflow streamlines. Versus in the case of larger particles, since the force of gravity is greater than the vertical component of the aerodynamic forces, they will settle on the ground or other surfaces^16,17^. Therefore, it can be concluded that the particle size, nature of the airflow field, environmental conditions, and emission source conditions affect the distance traveled by the particles.

In recent years, with the increase in the incidence of the COVID-19 pandemic, more research has been conducted to examine and assess the behavior of viral microorganisms in indoor environments^18–20^. These infections with small particles can occur through various routes, whether direct or indirect pathways involving the susceptible person^18,21^. The mucous membrane of the human respiratory system, as well as the mucous membranes of the eyes, nose, or mouth, have a favorable environment for the growth and reproduction of such microorganisms. Irritation of the mucous membranes of the laryngeal and nasal systems leads to coughing and sneezing, resulting in the release of large amounts of infectious particles within the process. Research results confirm that the rate of exposure to the virus is greatly affected by the rate of air exchange, airflow conditions such as relative humidity, temperature, location of ventilation systems, and injection source, especially in enclosed spaces^22–26^. Hand hygiene, keeping a physical distance, wearing a mask, and covering the face are known to be the primary tools in the fight against the SARS-CoV-2 virus. Branson et al.^27^ studied and determined the safe physical distancing for different respiratory events. They indicated that keeping a social distance is an important way to combat COVID-19. Some other non-pharmacological methods that can play a vital role in breaking the transmission chain are: creating physical barriers^28,29^, using ultraviolet inactivating lamps^30,31^ employing the air purifiers systems^32–34^, controlling the HVAC systems^26,35–37^ and so on.

Many studies have been carried out on the importance and role of the ventilation system in assessing the risk of disease transmission in the indoor environment. Anghel et al.^22^ proposed increasing the use of outdoor air and the rate of air change, decreasing the recirculation of air, and using high-efficiency particulate air filters to control and optimize the ventilation system and reduce infectious risk. A numerical study assessed the effects of relative humidity, wind speed, and social distancing on evaporative droplet transport in the outdoor environment^38^. The results revealed that under dry conditions, the cough-generated particle traveled distance could increase. Ahmadzadeh et al. investigated the influence of air conditioning on the dispersion of COVID-19-laden particles expelled during teachers speaking inside a classroom under different scenarios^39^. The results emphasize the significant effect of opening a window near the teacher in reducing the risk of student contamination. Tracking the distribution of infectious aerosols is extremely impacted by the airflow, and ventilation has a great performance in the discharging rate of coughed droplets from room^40^. In the other work, Yao and Liu^41^ assessed the effect of open window location on aerosol spreading in an enclosed bus. They concluded that the number of open windows in indoor environments increasingly affects the aerosol transmission and infection risk of individuals. Although most of this literature has evaluated the particle dispersion in a ventilated enclosure notwithstanding, there is a gap in knowledge between the influences of the different air conditioning settings and observing the safe social distance. To cover this gap, in the current study the effect of the input flow location, window opening frequency, and the height of the computational domain on the dispersion of the coughing particles and assessing the individuals' exposure to scattered particles via evaluating the fraction of the deposited particles on the individuals' face and suspended particles inside a social-distanced meeting room has been investigated.

Considering a large number of new infected people, deaths, mental and economic concerns, and uncertainties surrounding the COVID-19 epidemic suggest that more research on coronavirus transport dynamics in indoor spaces, such as meeting rooms and developing strategies for reducing virus transmission are vital. The present study uses the CFD technique to simulate the impacts of different air conditioning settings on the distribution of particles carrying the SARS-CoV-2 viruses released by the coughing of an infected person in a meeting room. Because the mucous membranes of the eyes, nose, and mouth are a favorable environment for the entry of microorganisms, to more accurately assess the individuals' exposure to these particles, the fraction of the particles deposited on the faces of individuals was examined separately. In particular, the aim is to study the effects of the window opening frequency, capacity of the exhausts, exhaust location, the direction of particle injection with inlet flow, and height of the room on dispersion and transmission of contaminants generated from an infected source to identify the safe zones and the most favorable scenario in a meeting room. This research can be significant because it helps the research community and regulatory agencies with the potential to increase a fundamental understanding of how environmental and human factors affect the transmission of the virus through the air from person to person.

## Materials and methods

**Figure 1:**
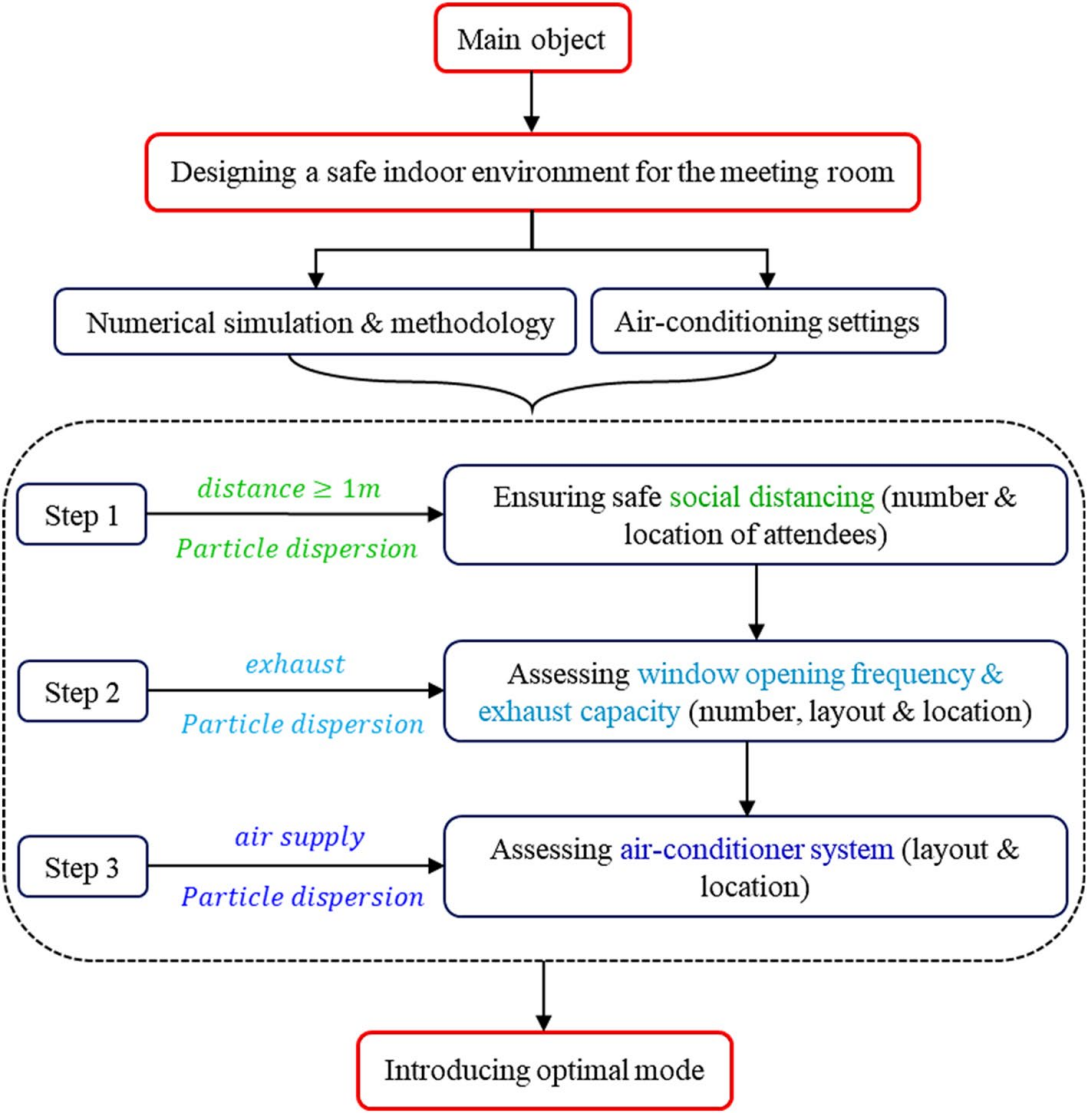
Flowchart of present study aimed at air-conditioned meeting room design for a safe indoor environment.

A comprehensive numerical work is performed to study the airflow and aerosols spreading in a mechanically ventilated meeting room, where fresh air enters the room through two air conditioner systems (ACS). The flowchart of this work is drawn in Fig. 1. First, steady-state simulation is solved using the Eulerian approach for airflow, then this solution is used as an input for the detection of unsteady-state distribution of particles based on the discrete phase model (DPM). Studying grid independence and validation of numerical simulation results based on literature works have been investigated. The following assumptions are adopted in this work: (1) the walls, seats, and desks are assumed to be adiabatic; (2) the head and body movements of the infected person during coughing have been ignored; (3) the droplets are assumed to be spherical particles with constant density. It is noted that most of the findings of the current study can be verified for other indoor environments such as classrooms.

### Design of the meeting room

**Figure 2:**
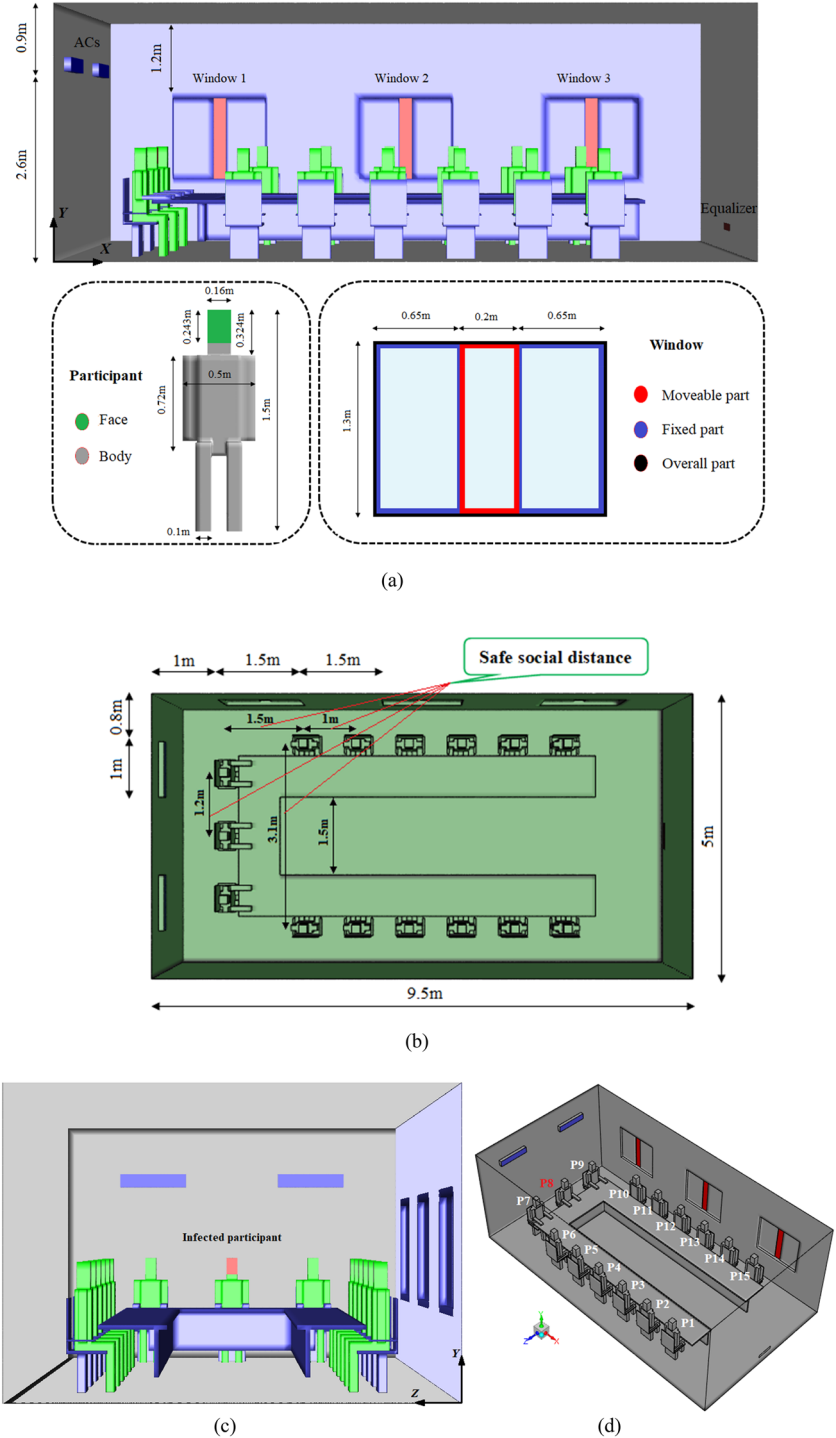
Schematics of the meeting room and sitting arrangement. (**a**) A side view of the computational domain and seated participants with a presentation of the different parts of the window; (**b**) dimensions of the room with a safe social distance (≥ 1 m); (**c**) front view of the computational domain and location of the infected participant (P8); (**d**) layout and naming of seated participants (the participants in the room are labeled P1 through P15).

In this study, a typical mechanically ventilated meeting room occupied by 15 seated participants is modeled. The dimensions of the simulation domain are 9.5 m (length) × 5 m (width) × 3.5 m (height) with a volume of approximately 165 m^3^, as shown in Fig. 2. Two wall-mounted air conditioning units are installed on the back or front wall side. There are 3 windows with an equalizer to exhaust the airflow located on the exterior and front wall side, respectively. In this study, the utilized window has consisted of a fixed area and a moveable area. As shown in Fig. 2a, the dimensions of the whole window are 1.5 m (X) × 1.3 m (Y), the moveable part is 1.5 m (X) × 0.2 m (Y), and the rest is used for daylighting (fixed part). The equalizer is assumed to be 0.2 m (Y) × 0.5 m (Z) at the height of 0.3 m from the floor. In addition, the meeting room consists of a U-shaped meeting table in the center of the room with equally spaced chairs around the table, more details about the size of the table have been presented in Fig. 2. The chairs have a total height of 1 m and the sitting height of 0.45 m. The side, top, front and isometric views of the room and the considered safe social distance are illustrated in Fig. 2a–d, respectively.

### Numerical model and boundary conditions

In this study, the commercial ANSYS Fluent 2021 software is utilized to solve the governing equations and numerical simulations. In this work, all the cases have been studied in summer conditions. Because the windows are directly exposed to outdoor airflow, we assumed their temperature to be 30 °C, as well as the surface of the inner walls was covered with insulating materials. The full-scale room is cooled with two air conditioner systems (1 m × 0.2 m and 2.6 m height from the floor) as velocity inlet boundary conditions with a relative humidity of 50% and a set temperature of 20 °C. The windows (1.3 m × 0.2 m, and 1 m height from floor) and an equalizer are exhausts that facilitate the air exit and have been modeled as a pressure outlet boundary condition with atmospheric pressure. A suitable air conditioning design to prevent poor indoor air quality while preserving an admissible energy consumption range depends on the minimum airflow rate, type, and location of air diffusers. To ensure that sufficient ventilation air is delivered to individuals, and to identify a minimum required supply airflow rate based on occupancy and load information, Anand et al.^42^ methods were adopted. As per Anand's work and ASHRAE standard 62.1^43^, the minimum required supply air rate is calculated as 1 m^3^/s. This is greater than the minimum requirement set by the current air conditioning system (0.7 m^3^/s).

**Figure 3:**
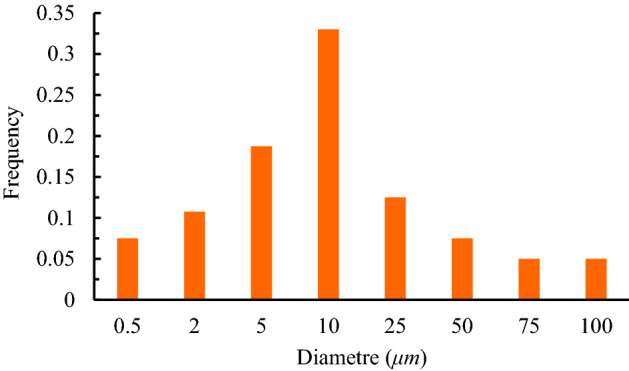
Distribution of particle diameter.

A no-slip adiabatic wall boundary condition is applied to all the walls and surfaces. In the current study, 15 participants in the room are labeled P1 to P15 as shown in Fig. 2d. Also, they are modeled by rectangular boxes instead of exact three-dimensional shapes to reduce modeling complexity as well as computational costs (Fig. 2a). In addition, considering that the participant marked with P8 in the session usually starts the discussion, this person has been selected as a hypothetical patient in the present study. It focuses more on introducing an optimal strategy for air conditioning settings, both air supply units, and exhausts, to reduce the exposure to indoor virus-laden particles. The area of a person's mouth of 0.03 m^2^, a stable coughing temperature of 37 °C, and the coughing average velocity of 11.2 m/s^10,44,45^ with a cough spread angle of 30°, respectively^45^ and their mass flow rates are calculated from Eq. (16). A total of 4000 particles ranging from 0.5 to 100 μm^46^ are ejected over a period of 0.5 s^47,48^ according to Fig. 3, which corresponds to a mass of 1.735 × 10^–7^ kg. The brevity of applied boundary conditions is tabled in Table 1. Moreover, the injected particles are assumed to be water liquid with a density of 998.2 kg/m^3^. Particles will be trapped when they contact the wall and surfaces or fall onto the ground.

**Table 1:** Summary of continuous and dispersed phases boundary conditions.

**Continuous phase**	
Inlet	Velocity inlet: 1.75 m/s; RH: 50%; density of air: 1.225 $\mathrm{g}/{\mathrm{m}}^{3}$; density of water vapor: 0.5542 $\mathrm{g}/{\mathrm{m}}^{3}$; supply temperatures: $20 ^\circ \mathrm{C}$; turbulent intensity: 5%; turbulent length scale: 0.035 m; dimensions: 100 cm $\times$ 20 cm; numbering: 2; escape
Outlet	Pressure outlet; ESCAPE
Wall	Windows: no-slip; $30 ^\circ \mathrm{C}$; participant: no-slip; $36 ^\circ \mathrm{C}$; other surfaces: no-slip and adiabatic; trap
**Dispersed phase**	
Expiratory flow	${v}_{inj}=11.2\mathrm{ m}/\mathrm{s}$; injection type: cone; injection angle: 30
Duration $(t)$; particles $(n)$; diameter ($\mathrm{\mu m})$	$t=0.5 s$; $n=4000$; (0.5, 2, 5, 10, 25, 50, 75, 100)

### Studied scenarios

Several scenarios are modeled to study the transport of respiratory droplets and their transmission in the room over time. These scenarios, as presented in Table 2, were simulated to fully investigate the effects of open or closed windows, their location, direction of the particle injection and inlet flow, and room height on particle spreading.

**Table 2:** Simulation scenarios.

Purpose	Scenario	Description	Sample graphical description
Effect of exhaust capacity	1	Window 1 open	
	2	Windows 1 and 2 open	
	3	All windows open	
	4	All windows close*	
Effect of exhaust layout	5	Windows 1 open	
	6	Windows 2 open	
	7	Windows 3 open	
	8	Windows 1 & 2 open	
	9	Windows 1 & 3 open	
	10	Windows 2 & 3 open	
Effect of air-conditioner location**	11 (mode 1)	AC on the back of P8; room height of 3.5 m	
	12 (mode 2)	AC on the back of P8; room height of 3 m	
	13 (mode 3)	AC on the front of P8; room height of 3.5 m	

*During this aim, the equalizer is the only outlet exhaust.

**During this aim, all the windows are assumed to be open.

### Mesh independence study

**Figure 4:**
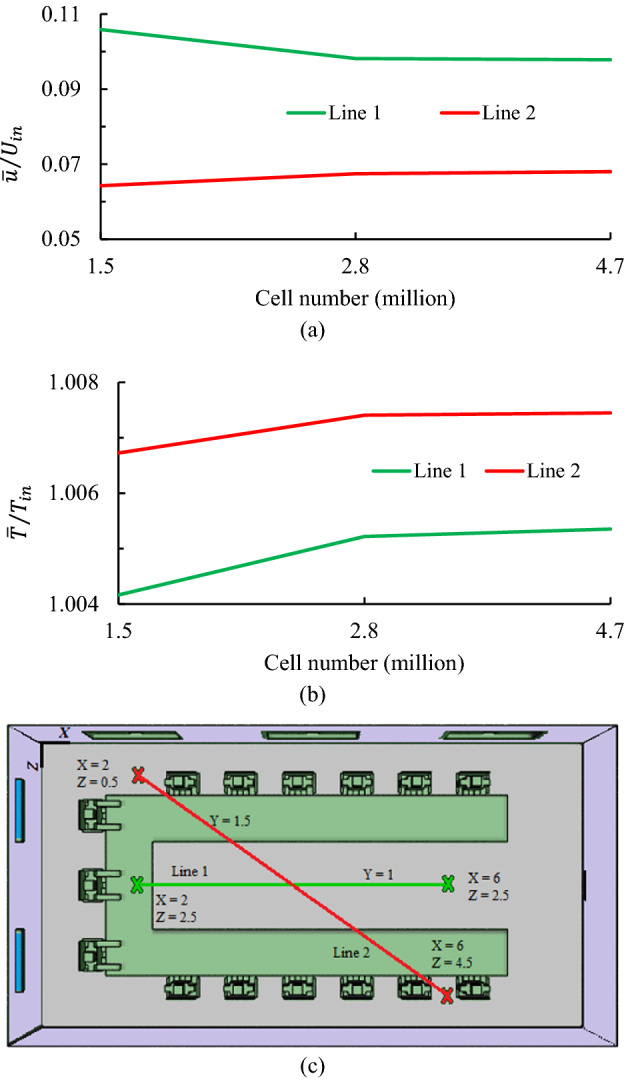
Grid sensitivity analysis for dimensionless: (**a**) airflow velocity ($\overline{u }/{U}_{in}$), and (**b**) airflow temperature ($\overline{T }/{T}_{in}$); (**c**) location of the investigated lines inside the domain.

Structural meshes with tetrahedral cells are used in this work. To ensure the results of the grid-compatible simulation, grid sensitivity analysis is performed by combining coarse, medium, and fine grids at the same boundary conditions. The cell numbers for these grids are 1.5, 2.8, and 4.7 million, respectively. The dimensionless airflow velocity ($\overline{u }/{U}_{in}$) and temperature ($\overline{T }/{T}_{in}$) values along the horizontal and diagonal lines of validation points inside the domain, that for each of the grids are shown in Fig. 4. Where, $\overline{u }=\frac{{u}_{max}+{u}_{min}}{2}$, $\overline{T }=\frac{{T}_{max}+{T}_{min}}{2}$ are the average velocity and temperature along the line, respectively. The difference between the results predicted by the medium and fine grids is not obvious (approximately less than 5%), and there is a compatibility agreement between them. Therefore, the medium grid case is selected for subsequent simulations.

### Governing equations

In this study, computational fluid dynamics was used to estimate the physics of the mixture of air and water vapor as a continuous phase and the droplets generated from the respiratory event as a discrete phase by applying the Eulerian–Lagrangian approach. The finite volume method is adopted to discretize the governing equations into algebraic equations. As well, to specify the airflow behavior near the wall surfaces, the standard wall function is employed. The semi-explicit method for pressure equations (SIMPLE) algorithm is employed to couple velocity fields and pressure, while the second-order upwind scheme is used for discretizing the convection and diffusion terms. The convergence of governing equations is assumed when the residuals are less than ${10}^{-4}$ for continuity and ${10}^{-6}$ for momentum and energy equations.

First, an incompressible, steady-state, and standard $k-\varepsilon$ model is solved using the Eulerian approach to attain the Reynolds-averaged Navier–Stokes (RANS) equations. Then, this solution is used as an input for unsteady particle tracking based on the discrete phase model (DPM) to investigate the dispersed flow of particles. Furthermore, to involve the Lagrangian stochastic effects, the discrete random walk (DRW) model is used. The governing equations for continuity, momentum, energy, and species transport, respectively, are given as:

1 $$\frac{\partial {\overline{u} }_{i}}{\partial {x}_{i}}=0$$

2 $$\rho {\overline{u} }_{j}\frac{\partial {\overline{u} }_{i}}{\partial {x}_{j}}=-\frac{\partial \overline{p}}{\partial {x }_{i}}-\frac{\partial {\tau }_{ij}}{\partial {x}_{j}}+\rho {g}_{i},\quad i,j=\mathrm{1,2},3$$

The Reynolds stresses $\left({\tau }_{ij}\right)$ is modeled by employing an eddy-viscosity approach,

3 $${\tau }_{ij}\approx \rho \left({\overline{{u}_{i}{u}_{j}}-\overline{u} }_{i}{\overline{u} }_{j}\right) ; {\tau }_{ij}=\frac{2}{3} \rho kI-{\mu }_{t}\left(\frac{\partial {\overline{u} }_{i}}{\partial {x}_{j}}+\frac{\partial {\overline{u} }_{j}}{\partial {x}_{i}}\right)$$

4 $$\frac{\partial }{\partial {x}_{i}}\left({\overline{u} }_{i}\left(\rho e+p\right)\right)=\frac{\partial }{\partial {x}_{i}}\left({\lambda }_{eff}\left(\frac{\partial T}{\partial {x}_{i}}+{\overline{u} }_{j}{\tau }_{ij}\right)\right)$$

In addition, the standard *k*–*ε* turbulence model is used for the modeling of *k*, the turbulent kinetic energy, and $\varepsilon$, the dissipation rate of turbulent energy, as described in Eqs. (5) and (6),

5 $$\frac{\partial \left(\rho k\right)}{\partial t}+\nabla \cdot \left(\rho \overrightarrow{u}k\right)=\nabla \cdot \left(\frac{{\mu }_{t}}{{\sigma }_{k}}\nabla k\right)+{G}_{k}-\rho \varepsilon$$

6 $$\frac{\partial \left(\rho \varepsilon \right)}{\partial t}+\nabla \cdot \left(\rho \overrightarrow{u}\varepsilon \right)=\nabla \cdot \left(\frac{{\mu }_{t}}{{\sigma }_{\varepsilon }}\nabla \varepsilon \right)+\frac{\varepsilon }{k}\left({C}_{1\varepsilon }{G}_{k}-{C}_{2\varepsilon }\rho \varepsilon \right)$$

where ${C}_{1\varepsilon }$, and ${C}_{2\varepsilon }$ are constants 1.44 and 1.92, respectively, and ${\sigma }_{k}$ and ${\sigma }_{\varepsilon }$ are 1.00 and 1.3, respectively. ${G}_{k}$ is the production of turbulence kinetic energy.

Eddy viscosity ${\mu }_{\tau }$ is expressed as

7 $${\mu }_{t}=\rho {C}_{\mu }\left(\frac{{k}^{2}}{\varepsilon }\right)$$

where ${C}_{\mu }$ is equal to 0.09.

In the DPM, the trajectory of a droplet, which is regarded as a particle, can be obtained by integrating Newton's second law, which balances inertia force with external forces acting on the droplets. The effect of gravitation force, drag force, lift force, and inertia force and the effects of Brownian motion (for droplets less than 5 µm) on the coupling between the discrete and continuous phases are also included in the simulation^49–51^. In a Lagrangian reference frame,

8 $${m}_{p}\left(\frac{d{u}_{p}}{dt}\right)={F}_{D}+{F}_{G}+{F}_{L}+{F}_{B}$$

These forces are calculated as follows

9 $${F}_{D}=\left(\frac{18{\mu }_{f}}{{\rho }_{p}{d}_{p}^{2}{C}_{c}}\right)\left({u}_{f}-{u}_{p}\right)$$

10 $${C}_{c}=1+\left(\frac{2\lambda }{{d}_{p}}\right)\left(1.257+0.4{e}^{-\left(1.1{d}_{p}/2\lambda \right)}\right)$$

11 $${F}_{L}=1.615{\rho }_{p}{\nu }^{0.5}{d}_{p}^{2}\left({u}_{f}-{u}_{p}\right)\left|\frac{d{u}_{f}}{dy}\right|sgn(\frac{d{u}_{f}}{dy})$$

12 $${F}_{G}=\left({\rho }_{p}-{\rho }_{f}\right)Vg$$

13 $${F}_{B}={m}_{p}G\sqrt{\frac{\pi {S}_{0}}{\Delta t}}$$

14 $${S}_{0}=\frac{216 \nu {k}_{B}T}{{\pi }^{2}{\rho }_{f}{d}_{p}^{5}{\left(\frac{{\rho }_{p}}{{\rho }_{f}}\right)}^{2}{C}_{c}} ,\quad {k}_{B}=1.3806452\times {10}^{-23} J/K$$

In the Eq. (13) G represents zero-mean, unit-variance, independent Gaussian random numbers, and $T$ is the absolute temperature of the fluid $(K)$.

Furthermore, in this model, the fluid velocity at the aerosol location is modeled by Discrete Random Walk (DRW) model as:

15 $${u}_{f}={\overline{u} }_{f}+{u}_{f}^{^{\prime}}={\overline{u} }_{f}+{\zeta } \sqrt{\frac{2k}{3}}$$

The mass flow rate of particles is presented as:

16 $$\dot{m}=\frac{4n\pi {\rho }_{p}{r}^{3}}{3t}$$

where $n$ is the total number of particles and $t$ is the coughing duration time. All simulations were performed using the standard $k-\varepsilon$ turbulent model, as this model has proven to be reliable in indoor airflow modeling and its low computational cost, as suggested by^52–54^. This turbulent model was described in detail in^55^.

### Validation

**Figure 5:**
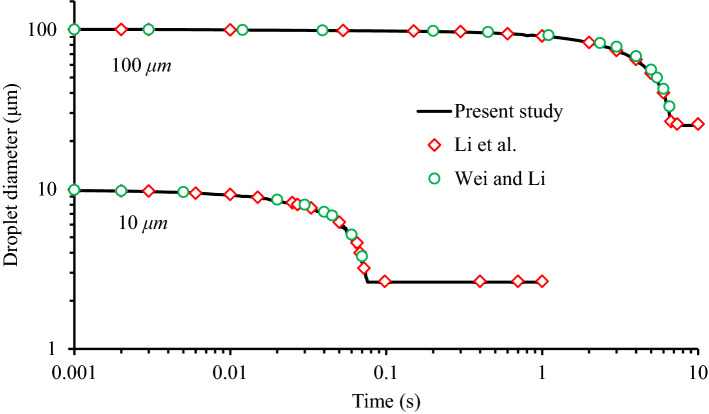
Result of the model validation using data from Li et al. and Wei and Li.

The efficacy of the model used in this work was checked by modeling the evaporation of a droplet in dry air for simulation of particle motion by^56,57^. Furthermore, to verify the continuous phase accuracy, the model was checked with the results of work by Ahmadzadeh et al.^39^ to verify the continuous phase, and after a good agreement, the solution results were implemented. As given following, the comparison verification results from Li et al. and Wei and Li have been presented. The study of Li et al. includes a computational domain as a three-dimensional (L × H × W = 4 m × 3 m × 2 m) enclosed room, where two size droplets of initial diameters of 10 and 100 μm at mass flow rates of 5.24 × 10^–11^ kg/s and 5.24 × 10^−8^ kg/s, respectively, evaporating at a constant temperature of 25 °C. Droplets consist of 98.2% water and 1.8% nonvolatile particles with a density of 1000 kg/m^3^. In this model, it was assumed that single droplets were released one after another with a time–frequency of 0.01 s at 37 °C. In this study, the multicomponent Eulerian–Lagrangian approach was employed to realize mechanistic modeling. For the simulation, the structured hexahedral method was used, which consists of over 600,000 elements. The predicted time-dependent diameter (solid lines) of droplets injected by coughing was compared against the theoretical results (circle and rhombus marks). As shown in Fig. 5, a satisfactory agreement can be observed between the numerical results of this study and the data reported in the literature. Besides, more details of the droplet evaporation model and equations have been provided in Appendix A-supplementary material.

## Results

In this section, the results of the various air conditioning settings on the distribution pattern of the indoor airflow and the particle dispersion are analyzed under different scenarios of exhaust capacity and location (window openings), and locations of the airflow supplies in the meeting room. First, the results of the continuous phase (indoor airflow) for several scenarios are presented, and then the dispersion of the particles and assessing the individuals' exposure to contaminant particles under the coughing respiratory exhaled particles are investigated. Furthermore, in the coughing case particle tracking time is assumed by 180 s to ensure that the final states of the majority of released particles have been determined.

### Results of continuous phase

The airflow inside the meeting room is the combined result of the forced convection source of the air conditioning system and the natural convection caused by the temperature gradient. Because the human body temperature is higher than the surrounding airflow, a small scale of vortices is observed around the human body (human thermal mass).

**Figure 6:**
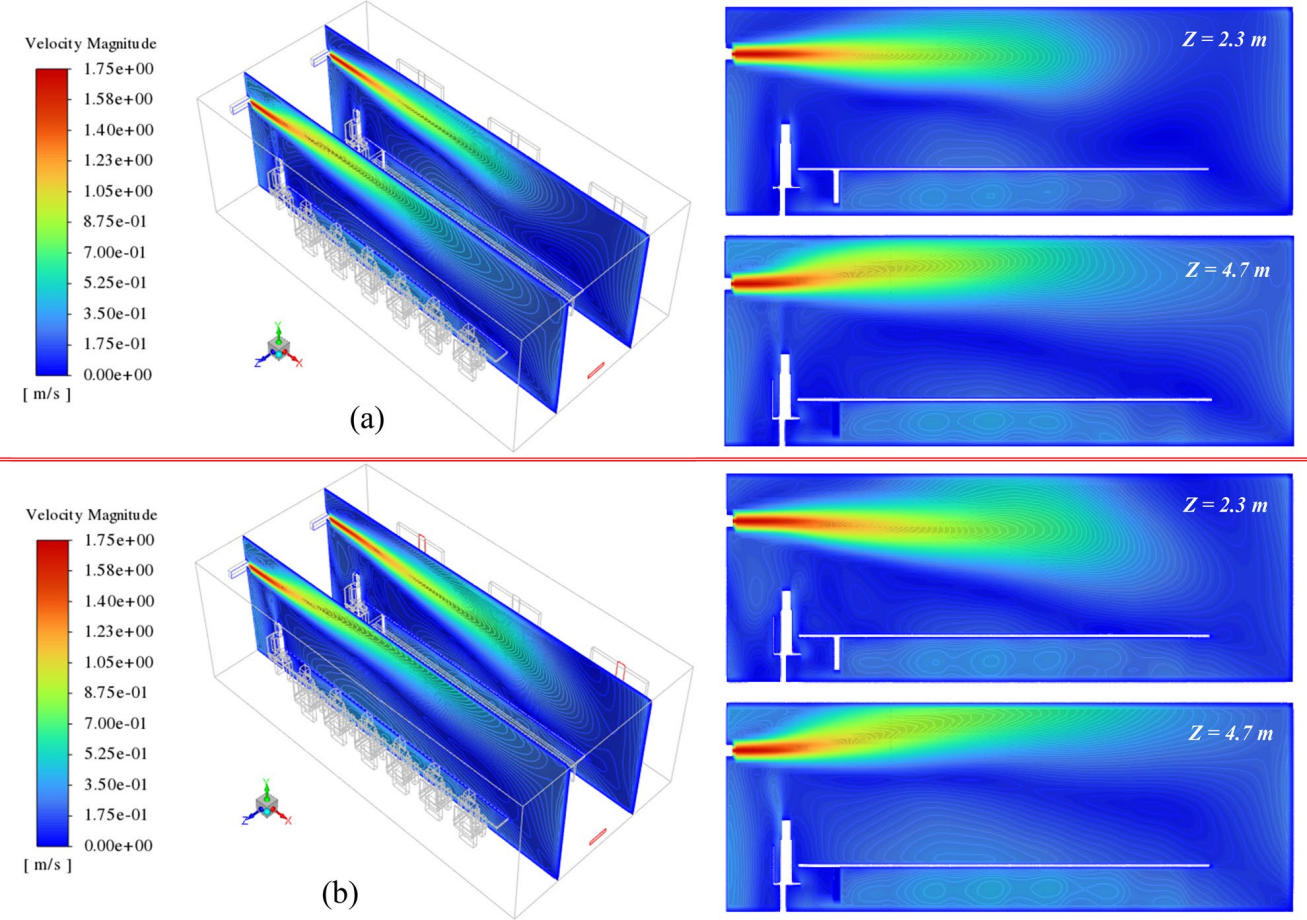
Velocity contour of indoor airflow at z = 2.3 and 4.7 m on the isometric view (left side) and XY plane (right side), for (**a**) all the windows, be closed, and (**b**) windows 1 and 3 be open.

**Figure 7:**
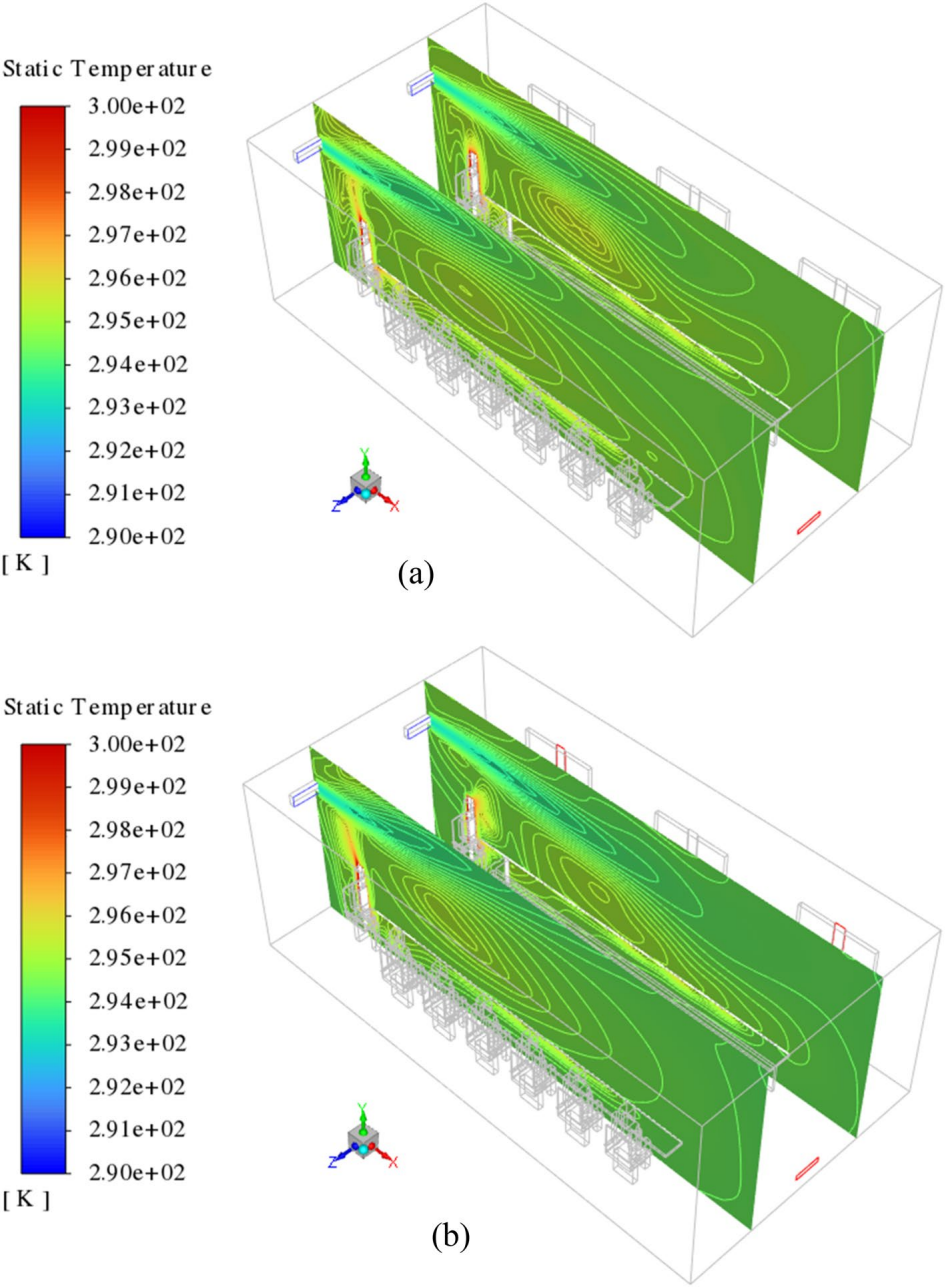
Temperature contour of indoor airflow at z = 2.3 and 4.7 m on the isometric view, for (**a**) all the windows, be closed, and (**b**) windows 1 and 3 be open.

The effect of the window opening (or capacity of exhaust) on the domain airflow velocity and temperature field are presented in Figs. 6 and 7, respectively. Figure 6 shows the velocity contours at Z = 2.3 and 4.7 m under the lack of open windows (Fig. 6a) and when windows 1 and 3 are added to exhaust capacity (Fig. 6b). As illustrated, in both scenarios, the airflow jets generated at the supplies can move directly across the domain to the exhausts. Furthermore, it can be seen that the addition of open windows affects the airflow in the domain and allows the airflow to have more exit paths, and as a result, the airflow can affect a larger region of the domain. This is inferred from the results presented on XY planes. These differences can drastically lead to changes in the fraction of suspended and deposited emitted particles. Similar effects also are seen in the temperature distribution contours. In the case where the equalizer is treated as only indoor airflow exhaust (Fig. 7a), the generated airflow jets travel just the small areas of the domain towards the equalizer and have less effect on the overall cooling of the environment. On the opposite of, adding the open window causes high scattering of the airflow, and consequently, more energy interchange occurs between the airflow molecules. This leads to observing relatively cold and desired conditions than the prior case (Fig. 7b).

**Figure 8:**
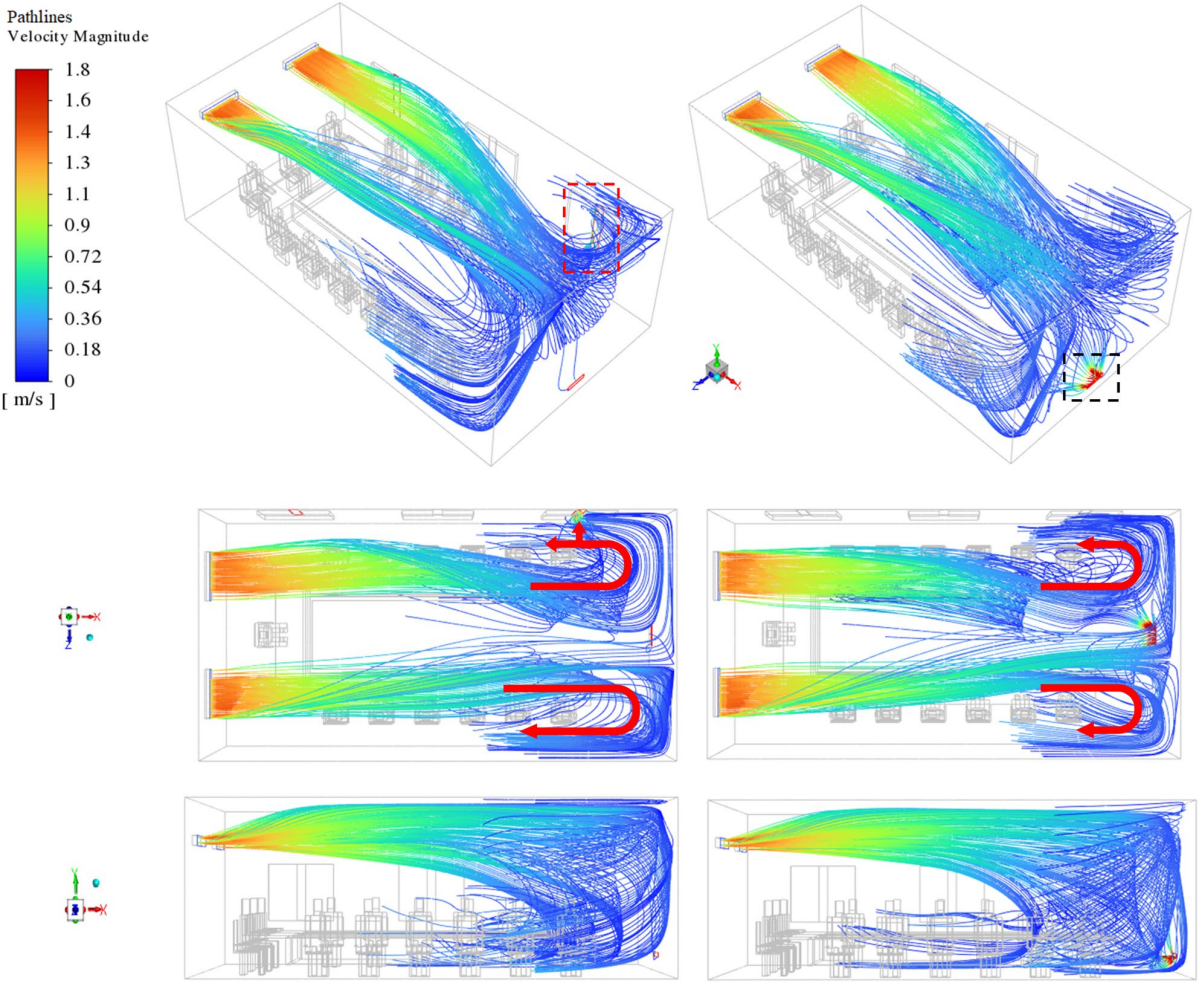
Generated velocity path lines inside domain from air supplies; left side: windows 1 and 3 be open, and the right side: all the windows, be closed.

Figure 8 illustrates the three views of the path lines of the velocity field from the air supply units for windows closed mode (right side) and opening mode of windows 1 and 3 (left side). In both scenarios, the airflows follow approximately the same paths after exiting the air supply units by approaching the exhausts. The airflow produced as soon it enters the domain moves towards the ceiling to reach the front wall and is then directed downwards. As expected, some of these paths leave the domain via the exhaust exits, while others turn in the opposite direction of the primary paths after colliding with the front wall and scatter inside the domain. By approaching the airflow paths to exhaust, the difference between the scenarios becomes more apparent. When all windows are closed, the concentration of path lines adjacent to the equalizer, as the only output path in this scenario, increases dramatically (black dashed bar in Fig. 8). Resulting in creating the relative symmetry between the inverse-path lines that can be seen nearing both sidewalls. In contrast, by opening the window(s) and increasing the airflow output capacity, the path lines tend to exit through the windows rather than equalize (red dashed bar in Fig. 8). For two main reasons, first, the window area is larger than the equalizer, and the windows are in a higher position relative to the equalizer, which leads to the approaching of the flow paths. Consequently, the created paths are asymmetric, unlike in the previous case. This behavior has been specified with the red arrows in Fig. 8.

**Figure 9:**
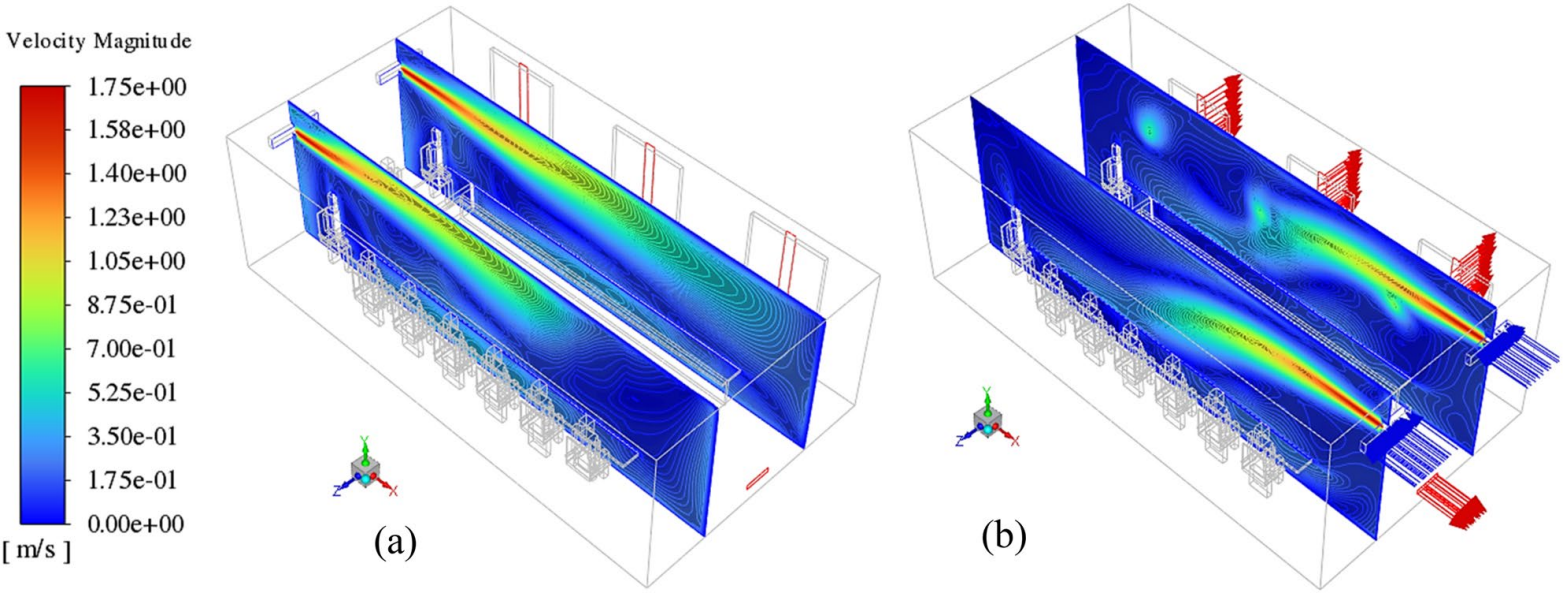
Airflow velocity contour at z = 2.3 and 4.7 m, and effects of the air supply location: (**a**) mode 2, and (**b**) mode 3, (blue and red arrow indicators point to input and output airflow, respectively).

The location of the air supplies is another important parameter that has a significant effect on the field of airflow velocity and thus the spread of viral particles. To clarify this influence, a comparison has been made in Fig. 9 between the height and direction of the air supplies. The most distinctive point is that the direct distance traveled by the produced jet stream in Fig. 9a is longer than in Fig. 9b. It can be attributed to the distance between the windows and the ceiling. It implies that by reducing the height of the room from 3.5 to 3 m, the distance between the windows and the produced jet of airflow is also decreased. And regarding the pressure and temperature difference between the open windows' surfaces and airflow streamlines and the resulting of tendency the streamlines to exchange momentum and energy, leads to observing this difference. Which causes a significant difference in the deposition and suspension of aerosols.

**Figure 10:**
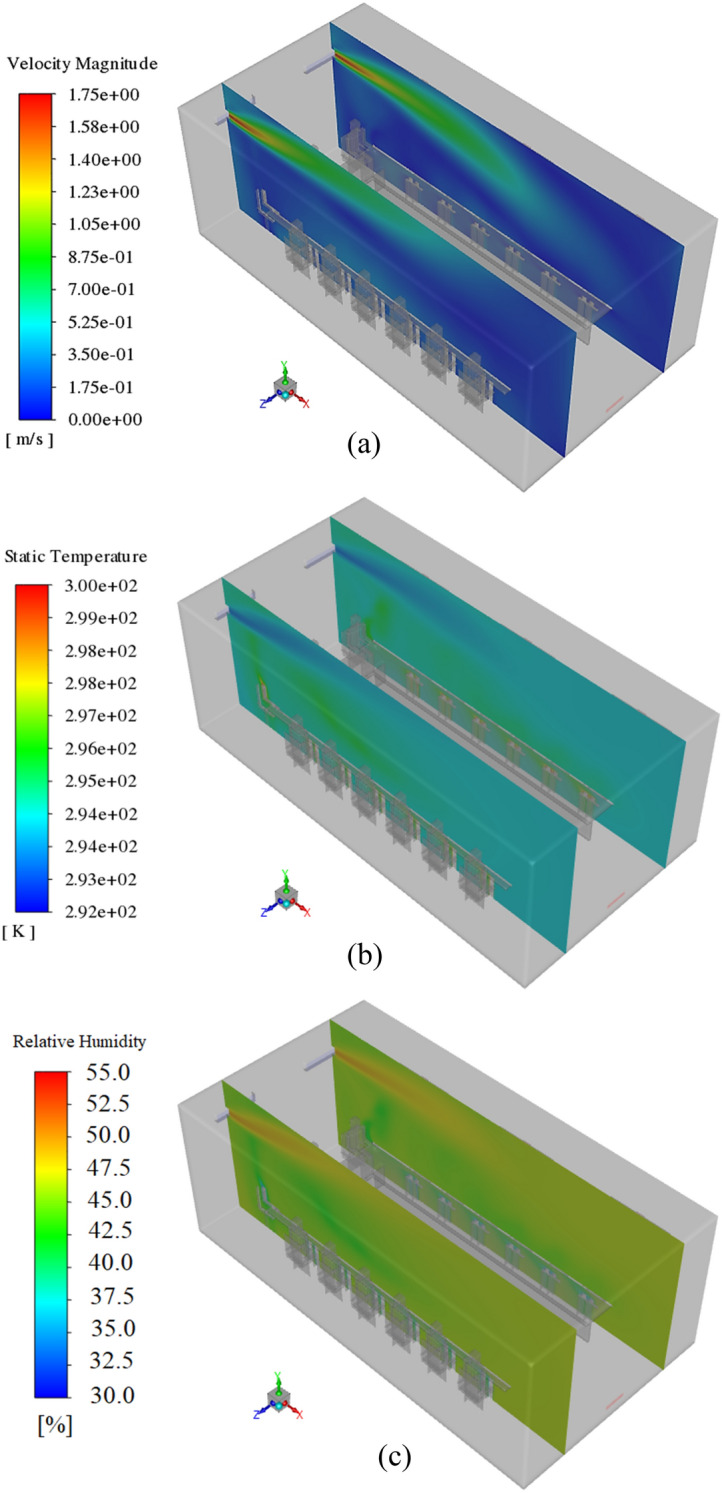
The isometric views of the velocity field, temperature field, and RH variations at Z = 2 and 4.9 m during scenario 1.

**Figure 11:**
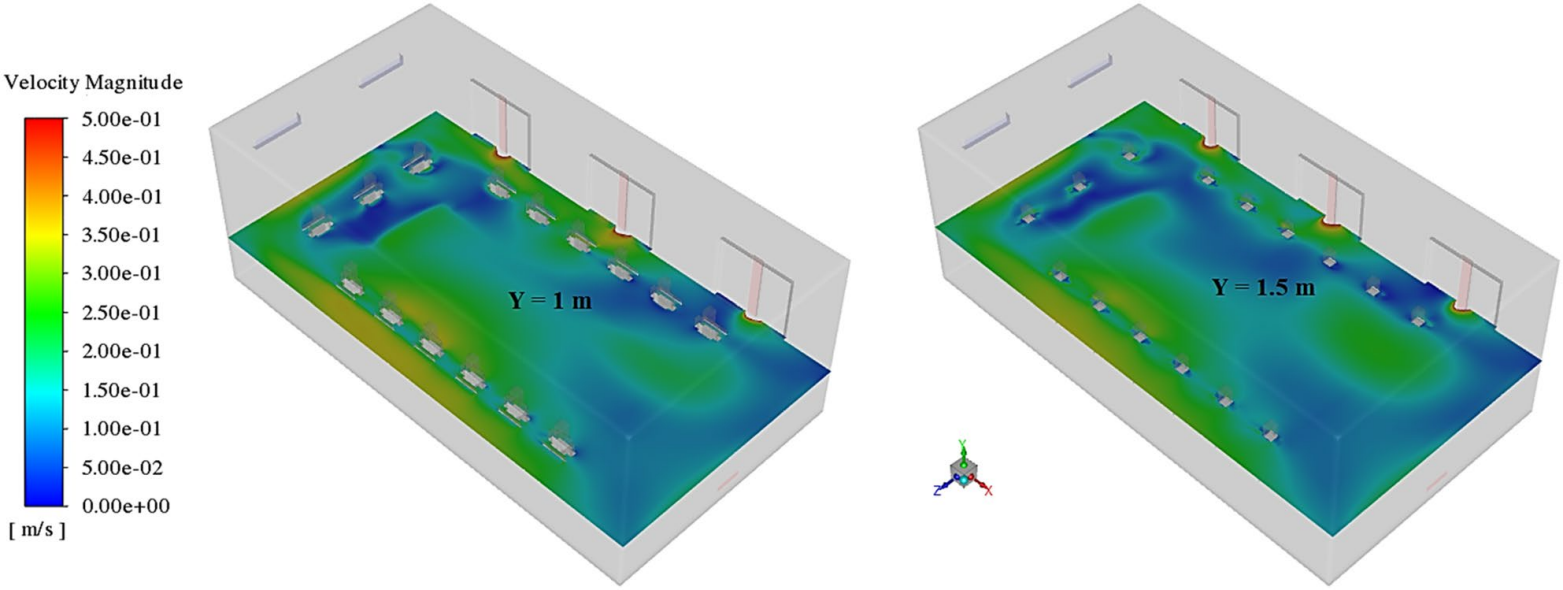
Velocity contours at Y = 1, and 1.5 m, in scenario 1.

Figure 10a–c show the isometric views of the velocity field, temperature field, and RH variations at Z = 2 and 4.9 m during scenario 1 (all windows are open). As illustrated, in the upper regions, the value of these three flow characteristics is higher than in the lower regions. This is due to the loss of momentum and energy of the incoming airflow, which occurs by moving to the lower parts. In Fig. 11 two different planes are selected to analyze the velocity field at Y = 1, and 1.5 m. As shown, the velocity field around people is such that it meets the needs of thermal comfort. However, the air velocity on the windows side is slightly slower than on the opposite side. It can be attributed to the decrease in the momentum and energy of the generated jets by exiting from the windows. It can be attributed to the decrease in the momentum and energy of the generated jets by exiting from the windows. And since the jet flow near the sidewall is far from the windows, they have a low chance to interchange the energy with the warm airflows around the windows.

### Results of dispersed phase

#### Effects of exhaust capacity

**Figure 12:**
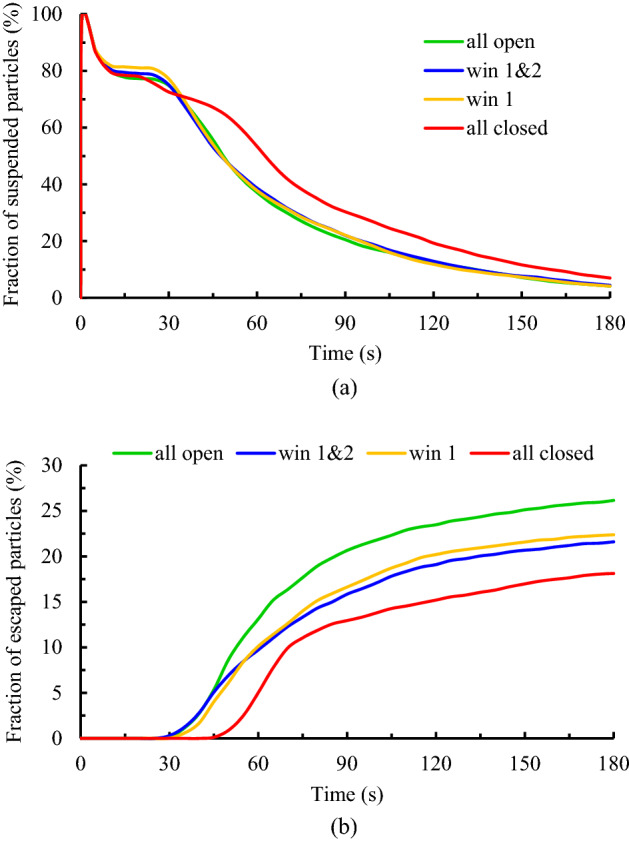
Effects of the exhaust capacity on the dispersion of the particles during various scenarios; (**a**) variation of the suspended particles versus time and (**b**) variation of the escaped particles versus time.

In this section, the effects of the capacity of the airflow exhausts, windows frequency, on the dispersion of the particles, and assessing the individuals' exposure to contaminant particles are evaluated by tracking the cough particles within 180 s inside the domain. Four scenarios are investigated to detect the final states of the emitted particles (Table 2). Figure 12a,b present the comparison of the fraction of the suspended and escaped particles versus the time, respectively. As shown in Fig. 12a, the reduction trend of the variation of the suspended particles over time is approximately independent of the airflow exhaust capacity. Although, when the equalizer is only the exit route of the particles from the domain (scenario 4), the shelf-life of the particles is relatively long. While there is no obvious difference between the results of the other three scenarios. Therefore, it can be concluded that increasing the capacity of the airflow exhaust in enclosed environments may be an effective strategy to reduce particulate matter. The results of Fig. 12b reveal the distinctive differences among the discussed scenarios. By adding the number of open windows (increasing the exhaust capacity), the rising trend of the escaped particles of the domain is improved. It is also worth mentioning that the comparison of the results between scenario 3 (maximum output capacity) and scenario 4 (minimum output capacity) indicates that there is a direct relationship between decreasing the output capacity and raising the suspended particle concentration in the room. In other words, the fraction of the escaped particles in the 180th second in scenario 3 is approximately 8% higher than in scenario 4. As shown in Fig. 12b, there is a 15-s delay in the exit of particle time in scenario 4, and this is because during this scenario all windows are closed and the particles have to travel a long distance to exit. As a result, it can be concluded that increasing the output capacity accelerates the particle exit process.

**Figure 13:**
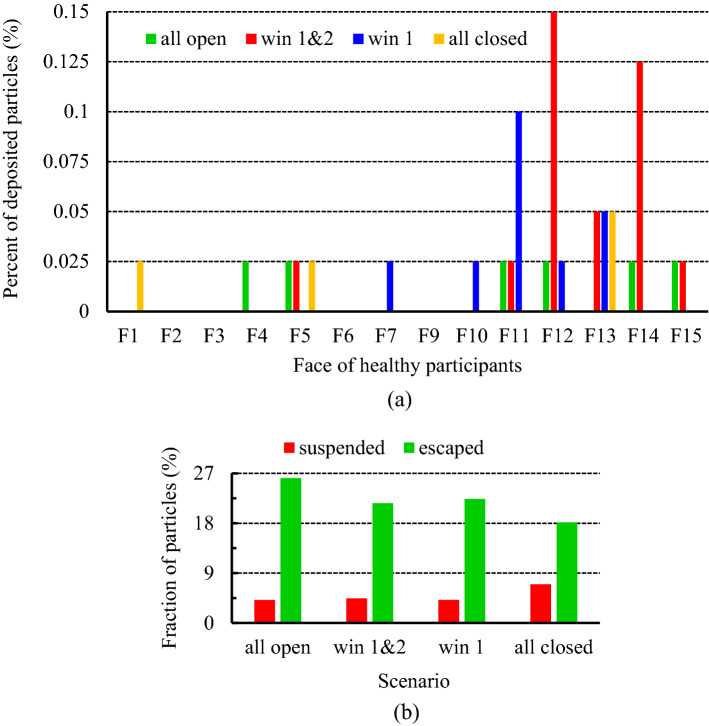
Effects of the exhaust capacity on the exposure risk of healthy participants during various scenarios; (**a**) the fraction of the particles deposited on the healthy individuals' faces, and (**b**) the fraction of the suspended and escaped particles at the end of particle tracking.

To better assess and detect the exposure risk of the healthy people in the room, the fraction of the deposited particles on their faces at the end of the simulation during the mentioned scenarios has been presented in Fig. 13a. Maybe at first glance, scenario 4 is chosen as the safest case in terms of the fraction of the deposited particles on the face. But this is not true. Because in determining the scenario with the lowest individuals' exposure to contaminant particles, two parameters play an important role: (1) the fraction of particles deposited on the face and (2) the quality of indoor air. Although the first item is the lowest in scenario 4, the fraction of the suspended particles is about 3% higher than in the other three scenarios (Fig. 13b). And that implies the air quality in this scenario is in the worst condition. Therefore, according to the above explanations and the results presented in Fig. 13a,b, it can be stated that people in scenarios 3 and 1, respectively, experience safer conditions. Another noteworthy point that can be inferred from Fig. 13a is that people who have sat on the windows side face a higher exposure risk than people on the opposite side. So that no particles have deposited on the faces of participants 2, 3, 6, and 9. It is due to that since the mass and momentum of the particles are low, airflow streamlines force the particles to follow their paths, resulting in a dramatic increase in the concentration of particles (or aerosols) around the exhaust. This, in turn, leads to people sitting near the exhausts experiencing unsafe conditions.

**Figure 14:**
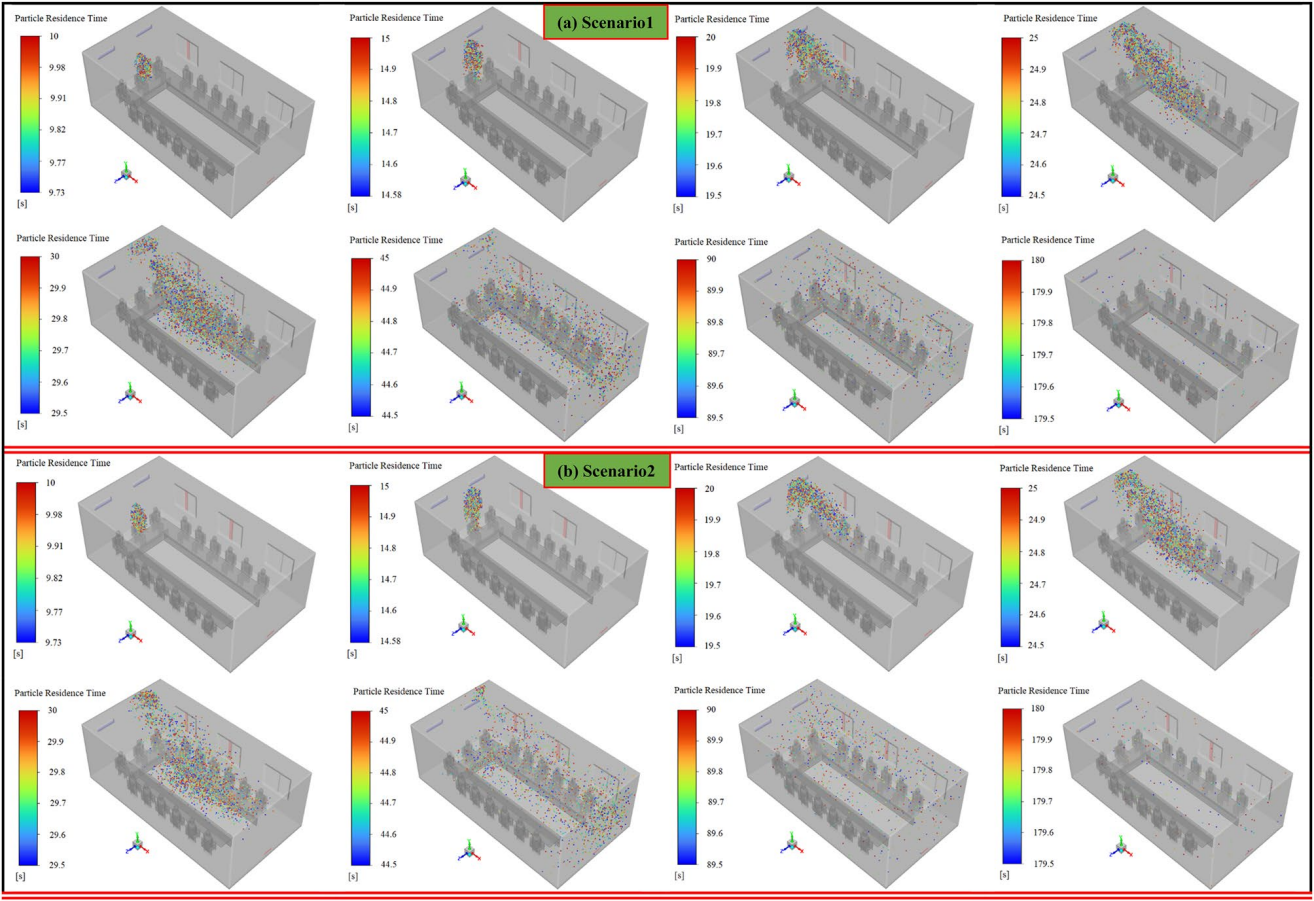

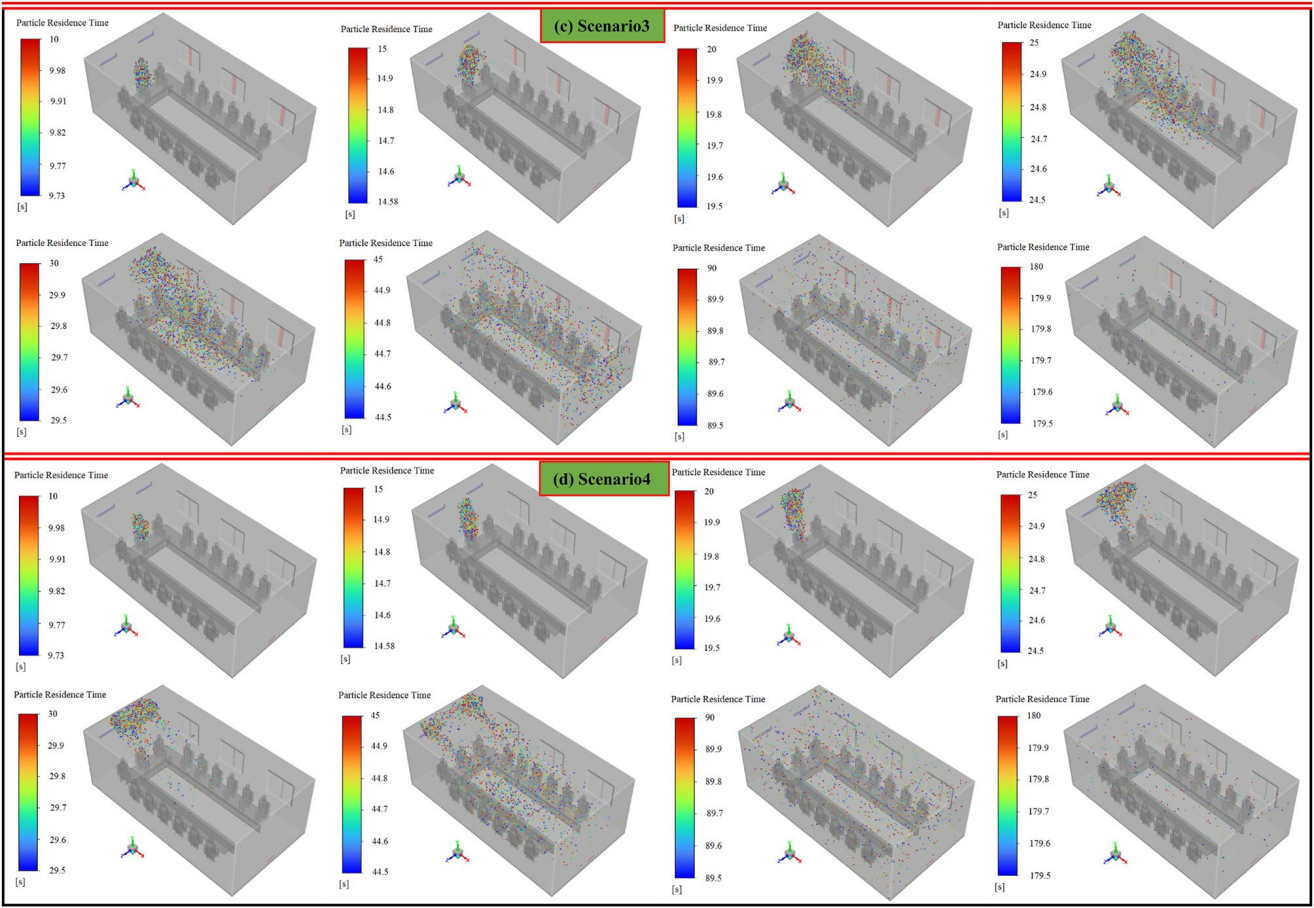
Aerosol cloud profiles at eight instances of time (10–180 s) inside the meeting room for different scenarios: (**a**) scenario 1, (**b**) scenario 2, (**c**) scenario 3, and (**d**) scenario 4.

In this section, four scenarios are considered to assess the behavior of the coughing-generated particles inside the meeting room. Figure 14 illustrates the aerosol-cloud profiles under the exhaust capacity impacts at eight instances of time (10–180 s) inside the meeting room for different scenarios. As expected and shown in Fig. 14, the capacity of the outputs strongly affects the transmission and spreading of particles inside the room. These factors can lead to symmetric (scenario 4) and asymmetric (scenarios 1, 2, and 3) spreading inside the environment. As illustrated, in general, dispersion patterns of the particles in the scenarios minimum of one of the windows is open, approximately similar. And increasing the open window(s) only accelerates the dispersion pattern. The main difference in the particle spreading is made between scenario 4 with three other ones. In scenarios 1, 2, and 3, the particles reach a wide dispersion stage after 20 s of injection, while in scenario 4, it occurs at the 30th second. Also, by opening the window (s), an asymmetric scattering pattern with more particles is observed next to the windows. In other words, opening more windows leads to most airflows moving towards them, and with the improvement of flow turbulence conditions, light particles have followed these dominant streams and spread in the domain. As time progresses, the particles scatter throughout the meeting room, by following the airflow streamlines. In Scenario 4, the particles reach the colony formation stage in the first 30 s, after which time, unlike in the previous case, begin to disperse symmetrically. Consequently, it can be said that closing all the windows delays the diffusion process and increases particle shelf-life. On the other hand, increasing the output capacity accelerates the particle spreading process inside the environment. To better understand this, particle diffusion animation in scenarios 3 and 4 is provided in supplementary videos, SV1–SV4.

#### Effects of exhaust layouts

**Figure 15:**
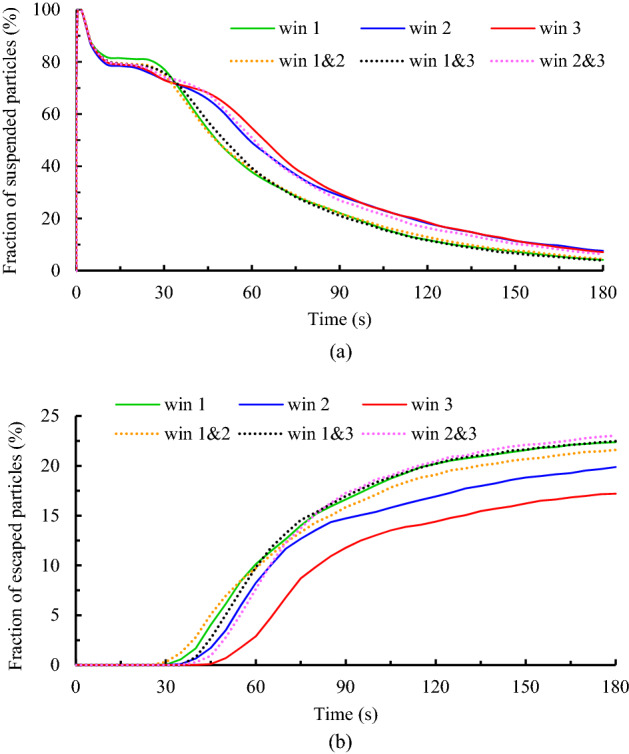
Effects of the exhaust layouts on the dispersion of the particles during scenarios 5 through 10; (**a**) variation of the suspended particles versus time and (**b**) variation of the escaped particles versus time.

To assess the effects of the airflow exhaust layouts on the dispersion of the particles and assess the individuals' exposure to contaminant particles, six scenarios (scenarios 5–10 from Table 2) have been studied. Similar to the previous section, a comparison is made between the fraction of the suspended and escaped particles during the scenarios. The results are provided in Fig. 15a,b. As shown in Fig. 15a, the variation of suspended particles' concentration inside the domain decreases over time so that its slope is greater for scenarios where the window near the diffusion source is open than for other scenarios. It means that opening the windows adjacent to the emission source has an efficient performance in decreasing the durability of the aerosols than opening the windows far from that. And that, in turn, reduces the individuals' exposure to contaminant particles. For instance, by changing the open window from window 2 to window 1, the fraction of the suspended particles at the same time over 3.5% decreases (7.6–4.15%). It goes back to the transport dynamics of the particles. When the window opens, most of the produced air jets tend to go out of domain through it. This tendency causes most of the particles, which have a slight momentum relative to the airflow, to move together with the airflow and thus scatter in amplitude. So, when the distance between the particle emission source and the open window is further, particles have more opportunity to scatter in the large area of the domain. On the other hand, because the aerodynamic force is greater than the gravitational force of the particles, the shelf life of the dispersed particles increases. This observation is slightly different for changes in the fraction of the escaped particles over time. As demonstrated in Fig. 15b, when only one of the windows opens (solid lines), similar to the suspended particles discussion, the nearest window to the emission source has the best performance in exiting the particles. And the difference between their efficiencies for particle exiting is relatively significant, at 22.5, 20, and 17.2 percent for scenarios 5, 6, and 7, respectively. While adding another open window increases performance. Among the scenarios with two open windows, in cases where windows 2 and 3 open (scenario 10), due to the direction of the incoming airflow, most of the particles accumulate around these windows, and as a result, the maximum fraction of escaped particles that occurs during this scenario (23%).

**Figure 16:**
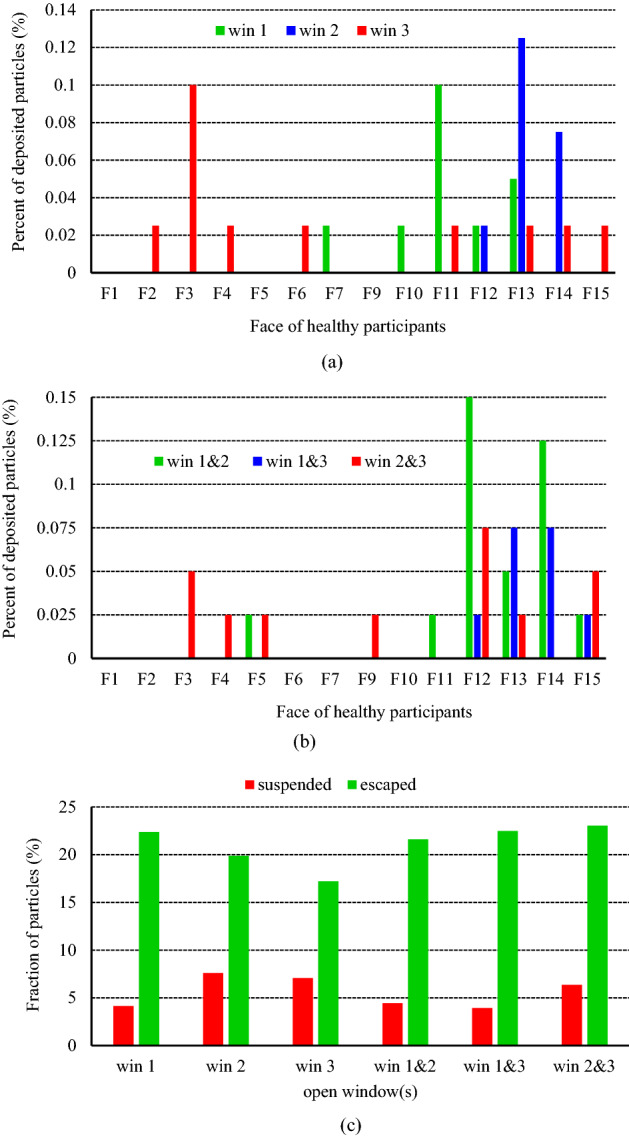
Effects of the exhaust layouts on the exposure risk of healthy participants during scenarios 5 through 10; (**a**) and (**b**) the fraction of the particles deposited on the healthy individuals' faces, respectively in scenarios 5, 6, 7, and 8, 9, 10, and (**c**) the fraction of the suspended and escaped particles at the end of particle tracking.

Figure 16 shows the fraction of particles deposited on the faces of healthy participants as well as the final state of the particles after 180 s in scenarios 5–10. According to the results of Fig. 16a,c, individuals present in scenario 5 face a lower exposure to contaminant particles in terms of the fraction of deposited particles on the face and air quality compared to scenarios 6 and 7. In addition, during these three scenarios, participants 1, 5, and 9 are at the lowest exposure to contaminant particles. A comparison of the results during scenarios 8, 9, and 10 reveals that opening windows 1 and 3 can be a suitable strategy to reduce the dispersion of the particles relative to opening windows 1 and 2 or 2 and 3. Meanwhile, similar to the conclusion of the previous section, the participants seated on the windows side are at the highest exposure to contaminant particles in all scenarios.

#### Effects of air-conditioner location

**Figure 17:**
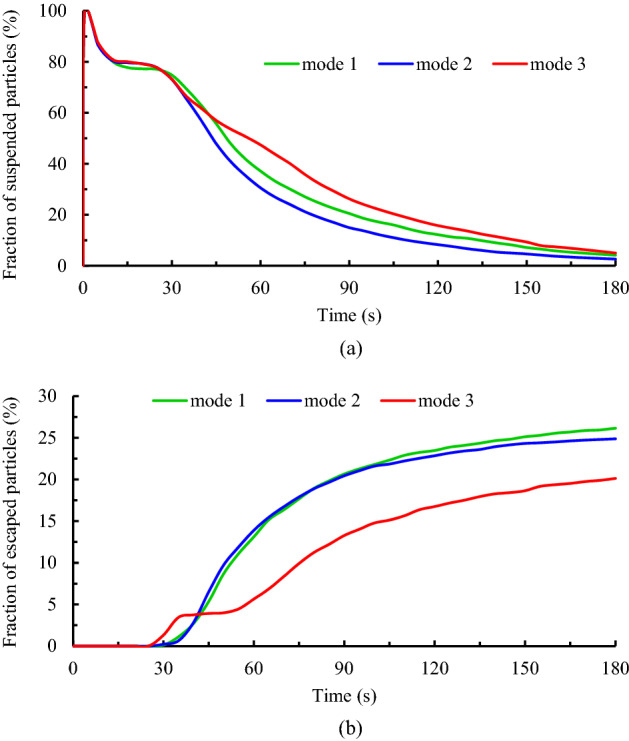
Effects of the air-conditioner location on the dispersion of the particles during scenarios 11 through 13; (**a**) variation of the suspended particles versus time and (**b**) variation of the escaped particles versus time.

**Figure 18:**
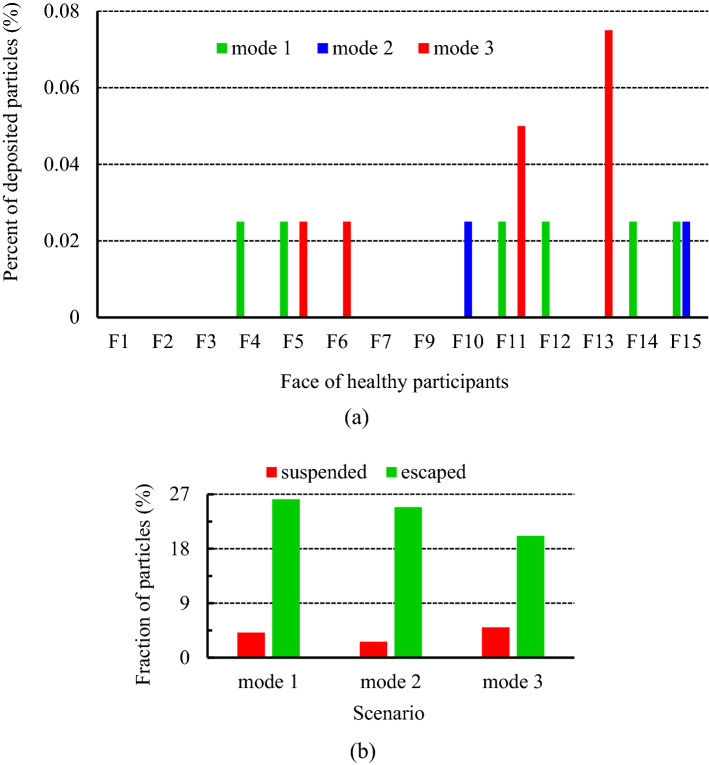
Effects of the air-conditioner location on the exposure risk of healthy participants during scenarios 11 through 13; (**a**) the fraction of the particles deposited on the healthy individuals' faces, and (**b**) the fraction of the suspended and escaped particles at the end of particle tracking.

**Figure 19:**
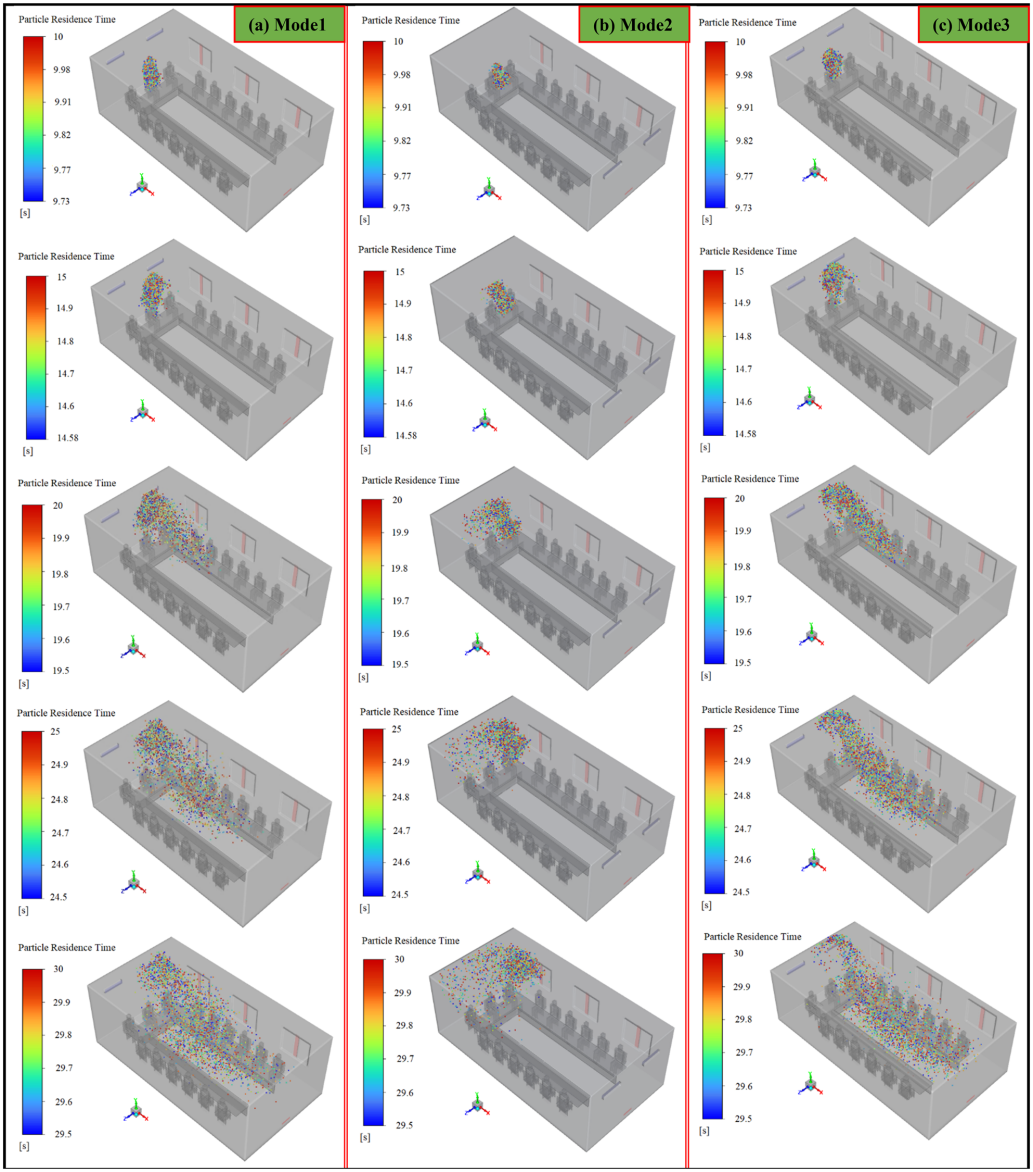

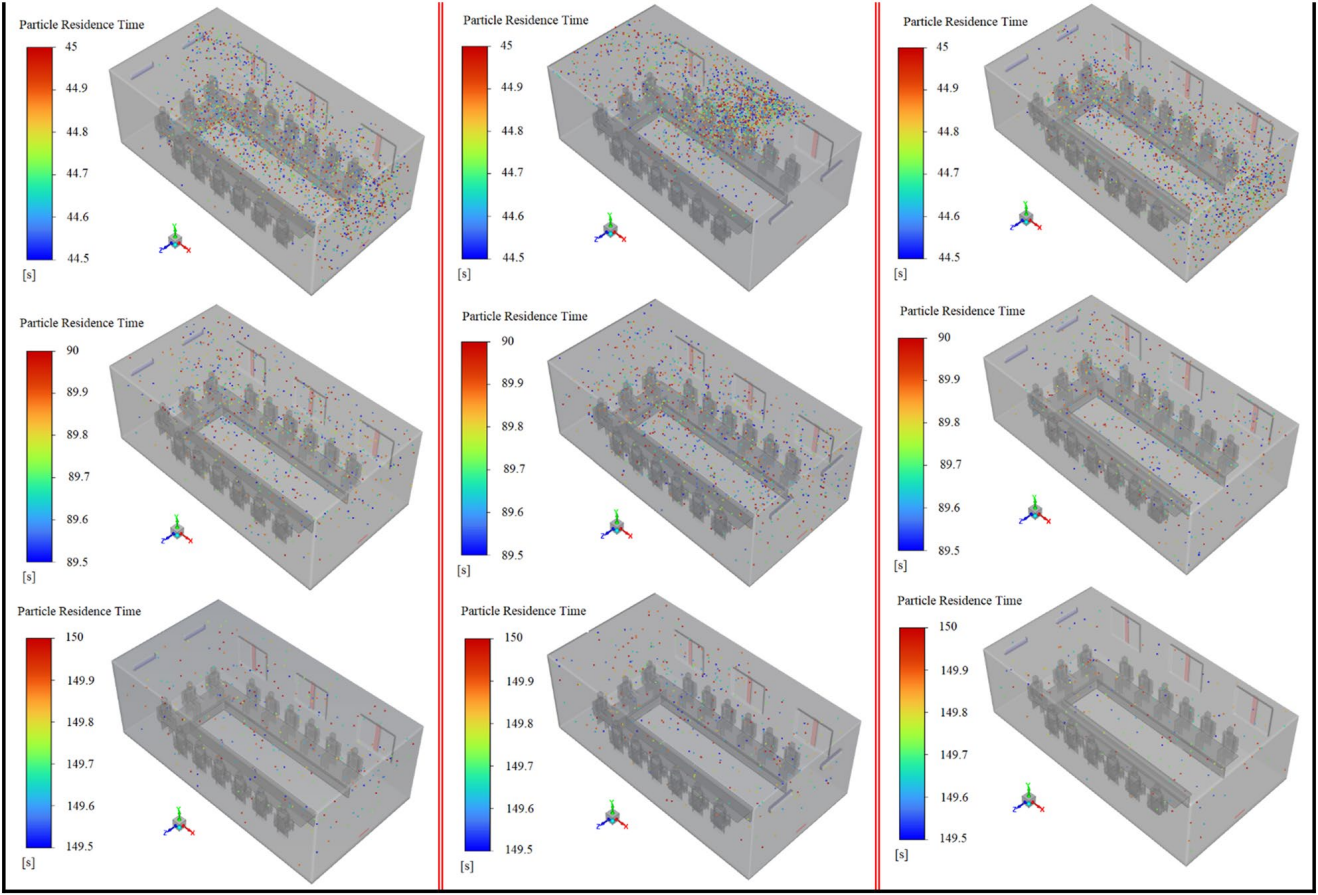
Aerosol cloud profiles at eight instances of time (10–150 s) inside the meeting room for different scenarios: (**a**) scenario 11 (mode 1), (**b**) scenario 12 (mode 2), and (**c**) scenario 13 (mode 3).

The installation location of air supply units in indoor environments is one of the other important factors that direct correlation with indoor air quality. In the present work, three scenarios, including mode 1, mode 2, and mode 3, have been selected to investigate the effects of airflow inlet on particle dispersion and exposure to contaminant particles. As well, to assess the effects of the height and direction of the inputs, it is assumed during these scenarios, all the windows are open. The results of the variation of the fraction of the suspended and escaped particles overtime during these scenarios are illustrated in Fig. 17a,b, respectively. As can be seen, by changing the direction of the air supply units and particle injection (in mode 3), meeting room air quality has descended both in terms of concentrations of the suspended and escaped particles. Because in this case, the generated airflow jets delay and limit the rapid dispersion of the particles and make it difficult to reach the exhaust zones, as shown in Fig. 19. Consequently, the shelf-life of the particles rises in mode 3 relative to mode 1 and mode 2. In addition, by reducing the distance between the airflow supply units and the ceiling (or room height), in mode 2, the final state improved slightly, especially the fraction of suspended particles. So that the fraction of the suspended and escaped particles after 180 s in mode 2 are 2.63% and 25%, respectively, and in mode 3 are 5% and 20%, respectively (Fig. 18b). As a result, it can be concluded that decreasing the distance between the airflow supply units and the ceiling rather than shifting the input airflows relative to the emission source direction is a safer and cleaner strategy for making suitable indoor air quality conditions. These observations are also verified by the results of Fig. 18a,b. The number of participants in mode 2 who are directly involved with viral particles is less than in the other modes (2 versus 6 and 4 participants in modes 1 and 3, respectively). This behavior can be attributed to the nature of the aerosols and the short distance between the windows and airflow supplies with the ceiling in this mode. Aerosols are very light and rise with minimal resistance to aerodynamic force and follow airflow streamlines. This results in significant differences between the aerosol clouds created during these scenarios, as shown in Fig. 19a–c. For better evaluation of the particle transports, the animation of the dispersion of the particles in these modes is presented in supplementary videos, SV5–SV7.

## Discussion

Regarding that, people spend over 80% of their lives in indoor environments. Thus it is necessary to study and adopt more effective strategies against pathogens, such as the SARS-CoV-2 virus in enclosed and crowded spaces. One of the most effective and desired strategies is employing cost-effective air-conditioning interventions, to improve air distribution performance and reduce the transmission of infectious diseases. This study focuses on the spreading of virus-carrying particles in a building environment that may have high potential exposure to contaminant particles. This study showed that a healthy and safe indoor environment can be provided by redesigning the installation location of the air-conditioner systems, observing the appropriate social distance, using an optimal open window(s) layout, and reconsidering the airflow.

According to the results obtained and the discussions presented in the above sections, it can be found that reducing the height of the room, scenario 12, has the greatest impact on improving the air quality of the room. Therefore, the risk of people suffering from both in terms of the fraction of suspended particles and trapped on the face is the lowest compared to other conditions studied. In addition, while all the windows are closed, although the number of exposed individuals is relatively low, the air quality in the room is not good in terms of airborne particles. On the other hand, among all the studied scenarios, healthy people are confronting the highest exposure to contaminant particles in scenario 9. As a result, it can be suggested that opening the windows, especially the windows located close to the emission source, when the distance between the airflow supplies (or even open windows) with the room ceiling is in the minimum state, not only speeds up the particle exit process but also reduces the number of exposed people.

The limitations of this study should also be considered. This study focuses on the spread of exhaled droplets by a cough jet by only one participant. However, the present model allowed us to study the effects of various scenarios in a detailed manner. In this study, we have not considered the movement of people during the simulation, and this causes a difference in airflow and thus the scattering of particles. In addition, for simplicity, we assumed the door closed during the simulation.

## Conclusion

This work uses the computational fluid dynamic method to investigate the flow dynamics and dispersions of particles of different sizes emitted by coughing a COVID-19-infected person in an observed safe social distance meeting room. 3D simulations were carried out to explore the characteristics of the airflow field and the impacts of the capacity of the airflow exhausts (numbering the open windows), open windows layout, and location of the airflow supplies on the spread of virus aerosol particles. This study can provide a safe reference for the design and construction of buildings. In conclusion, this study provides the mechanism for the dispersion of infectious aerosols, and the possibility of COVID-19 exposure to contaminant particles by air conditioning systems. It also highlights important recommendations regarding the selection of the best position of the airflow inputs and outputs in a heating, ventilation, and air conditioning (HVAC) system. Several conclusions may be drawn from the data presented in this study, as follows:

1. The conclusions made in this work show that opening the window(s) in indoor environments has a significant effect on air quality. As well, the exposure to contaminant particles for healthy people in the meeting room strongly depends on the distance of the open window(s) with the patient.
2. Actions such as decreasing the distance between the airflow supply units and ceiling, increasing the capacity of outputs, and use of the equalizer can provide important recommendations for disease control and optimization of ventilation in indoor environments.
3. Reducing the distance between the ventilation systems installed location and the ceiling can decrease the fraction of the suspended and escaped particles by over 2.5% and 5%, respectively, and at-risk individuals from 6 to 2 people.
4. Opening adjacent windows of the emission source has a better performance against the exit of particles from the environment than the window(s) that is farthest from that.
5. Reducing the output capacity not only increases the concentration of suspended particles but also increases the traveled distance by particles inside the domain.
6. As well, the results demonstrated when the direction of input airflow and generated particles were the same, the fraction of suspended particles of 4.125%, whereas if the inputs were shifted to the opposite direction of particle injection, the fraction of particles in fluid increased by 5.000%.
7. It has been found that in almost all studied scenarios, participants sitting near the exhaust (open windows) are at a higher risk of being infected by contaminated particles. This is because the motion of the particles is dominated by high-momentum airflow streamlines and subsequently low-inertia particles together with the airflow leave the domain via the exhaust.
8. As the distance between the infected person and the open window increases, at the same exhaust capacity, the number of people at the exposure to contaminant particles increases.

Furthermore, the simulation results raise concerns due to the long residence times of aerosols and traveled distances that exceed social distancing guidelines by reducing the output capacity. It should be noted that proceedings such as decreasing the distance between the airflow inlets and ceiling, decreasing recirculation of air, increasing the capacity of outputs, and using the equalizer are considered important prevention measures. Wearing masks and keeping social distance are important health ways that people can contribute to breaking the transmission chain of disease. This research also presents an optimal strategy, offers some recommendations in specifying desirable air conditioning control methods, and can minimize particle dispersion and exposure to contaminant particles.

## Supplementary Information

Supplementary Information 1.

Supplementary Information 2.

## Data Availability

The datasets used and/or analyzed during the current study available from the corresponding author on reasonable request.

## Abbreviations

ACS: Air conditioner systems
CFD: Computational fluid dynamic
COVID-19: Corona Virus Disease-2019
DPM: Discrete phase model
DRW: Discrete random walk
HVAC: Heating, ventilation, and air conditioning
PT: Public transportation
P: Participant
RANS: Reynolds-averaged Navier–Stokes
RH: Relative humidity
SARS-CoV-2: Severe Acute Respiratory Syndrome Coronavirus 2
SIMPLE: Semi explicit method for pressure equations
Win: Windows

## Parameters

$${d}_{p} (\mathrm{m}$$): Particle diameter
$$e (\mathrm{J}$$): Energy
$${F}_{B} (\mathrm{N}$$): Brownian force
$${F}_{D} (\mathrm{N}$$): Drag force
$${F}_{G} (\mathrm{N}$$): Gravitational force
$${F}_{L} (\mathrm{N}$$): Lift force
${g}_{i} (\mathrm{m}/{\mathrm{s}}^{2}$): The gravitation acceleration in the ${x}_{i}$ Direction
$${k}_{B} (\mathrm{J}/\mathrm{K}$$): Boltzman constant
${\dot{m}} (\mathrm{kg}/\mathrm{s}$): Rate of change of droplet mass
$${m}_{p} (\mathrm{kg}$$): Particle mass
$$p (\mathrm{Pa}$$): Pressure
$$r (\mathrm{m}$$): Particle radius
$$T (\mathrm{K}$$): Temperature
$${T}_{in} (\mathrm{K}$$): Inlet temperature
$$\overline{T } (\mathrm{K}$$): Average temperature
$$t (\mathrm{s}$$): Time
$V ({\mathrm{m}}^{3}$): Aerosol (also control) volume
$${U}_{in} (\mathrm{m}/\mathrm{s}$$): Inlet velocity
$$\overline{u } (\mathrm{m}/\mathrm{s}$$): Average velocity
${u}_{i} (\mathrm{m}/\mathrm{s}$): Flow velocity in the ${x}_{i}$ direction
${u}_{f}^{^{\prime}} (\mathrm{m}/\mathrm{s}$): Fluctuating eddy velocity
${u}_{f} (\mathrm{m}/\mathrm{s}$): The velocity of the fluid at the aerosol location
$${u}_{p} (\mathrm{m}/\mathrm{s}$$): Particle velocity
$$x (\mathrm{m}$$): Fluid location
$${x}_{p} (\mathrm{m}$$): Particle location
${C}_{c}$: Cunningham correction factor
${C}_{\mu }$: $k-\varepsilon$ Model empirical constant
$$sgn$$: Sign function

## Greek symbols

$${\rho } (\mathrm{kg}/{\mathrm{m}}^{3}$$): Density
${\rho }_{f} (\mathrm{kg}/{\mathrm{m}}^{3}$): Density of the fluid
$${\rho }_{P} (\mathrm{kg}/{\mathrm{m}}^{3}$$): Particle density
${\mu }_{f} (\mathrm{Pa s}$): Fluid dynamic viscosity coefficient
$${\mu }_{t} (\mathrm{Pa s}$$): Eddy viscosity
$\lambda (m$): Mean free path
${\lambda }_{eff} (\mathrm{W}/\mathrm{mK}$): Effective thermal conductivity
$${{\tau }_{ij}} (\mathrm{Pa}$$): Reynolds stresses
$\varepsilon ({\mathrm{m}}^{2}/{\mathrm{s}}^{3}$): Turbulence dissipation rate
$\nu ({\mathrm{m}}^{2}/\mathrm{s}$): Fluid kinematic viscosity coefficient
$$\pi$$: Pi number ($$\approx 3.14$$)
$\zeta$: Normal random number
