# Supplementary material for: A numerical approach for preventing the dispersion of infectious disease in a meeting room
Source: Sci Rep. 2022 Oct 10;12:16959. doi: 10.1038/s41598-022-21161-z (PMC9549042; doi:10.1038/s41598-022-21161-z)
Supplement: Supplementary file 1 — Supplementary Information 1. [file 41598_2022_21161_MOESM1_ESM.docx]

**Appendix A**

**A numerical approach for preventing the dispersion of infectious disease in a meeting room**

Mahdi Ahmadzadeh^1^, Mehrzad Shams^1^*

^1^Faculty of Mechanical Engineering, K.N.Toosi University of Technology, Pardis St., Vanak Sq., Tehran, Iran

* To whom all correspondence should be addressed, [shams@kntu.ac.ir](mailto:shams@kntu.ac.ir)

**Droplet evaporation model**

Droplet evaporation model equations that were used in validation (section 2.6) are discussed in this appendix. Droplet evaporation is modeled by diffusive molar flux of water vapor droplet into the air, $N_{i}$, via the following expression

| $N_{i}=k_{c}\left( C_{i,d}-C_{i,\infty} \right)$ | (A.1) |
| --- | --- |

where $N_{i}$ is evaporative molar flux of vapor,$k_{c}$ is mass transfer coefficient, $C_{i,d}$ and $C_{i,\infty}$ are vapor concentration at the droplet surface and surrounding medium, respectively. In this case, $C_{i,d}$ is related to the saturated vapor pressure via:

| $C_{i,d}=\frac{P_{sat}}{RT_{d}}$ | (A.2) |
| --- | --- |

where R is the universal gas constant and $T_{d}$ is droplet surface temperature. $C_{i,\infty}$ is then related to the partial vapor pressure,

| $C_{i,\infty}=x_{i}\frac{p}{RT_{\infty}}$ | (A.3) |
| --- | --- |

where $x_{i}$ is the local mole fraction of species $i$, $p$ is local pressure, and $T_{\infty}$ is bulk temperature of the surrounding continuous phase. The mass transfer coefficient from droplet to the surrounding continuous phase, $k_{c}$, is correlated with the Reynolds number and the Schmidt number by the following expression:

| $k_{c}=\frac{D_{i,m}}{D_{d}}\left( 2.0+0.6Re^{0.5}{Sc}^{0.33} \right)$ | (A.4) |
| --- | --- |

where $D_{i,m}$ is the diffusion coefficient of vapor in the continuous phase.

The change in mass of the droplet due to evaporation at every time step obtains as

| $m_{d}\left( t+\Delta t \right)=m_{d}\left( t \right)-N_{i}A_{d}M_{w,i}\Delta t$ | (A.5) |
| --- | --- |

where $m_{d}$ is the droplet mass, $M_{w,i}$ is the molecular weight of species $i$, and $A_{d}$ is the droplet surface area.

A simple heat balance equation which conveys the relationship heat transfer between the droplet and the continuous phase,

| $m_{d}c_{p}\frac{dT_{d}}{dt}=hA_{d}\left( T_{\infty}-T_{d} \right)-\frac{dm_{d}}{dt}h_{f\mathsf{g}}$ | (A.6) |
| --- | --- |

where $c_{p}$ is the specific heat of droplet, ${dm_{d}}/{dt}$ is evaporation rate, and $h_{f\mathsf{g}}$ is latent heat of droplet. The convective heat transfer coefficient, $h$, is calculated as,

| $h=\frac{\lambda_{a} ln\left( 1+B_{T} \right)}{D_{d}B_{T}}\left( 2.0+0.6Re_{d}^{0.5}{Pr}^{0.33} \right)$ | (A.7) |
| --- | --- |

where $Pr$ is the Prandtl number and $\lambda_{a}$ is the thermal conductivity of air. $B_{T}$ is the Spalding heat transfer number,

| $B_{T}=\frac{C_{pv}\left( T_{a}-T_{d} \right)}{h_{fg}-\frac{q_{d}}{\dot{m}_{d}}}$ | (A.8) |
| --- | --- |

where $\dot{m}_{d}$ is the droplet evaporation rate and $q_{d}$ is the heat energy transferred to the droplet, and $C_{pv}$ is the specific heat of the droplet vapor.
